# Component stability in low-space massively parallel computation

**DOI:** 10.1007/s00446-024-00461-9

**Published:** 2024-02-08

**Authors:** Artur Czumaj, Peter Davies-Peck, Merav Parter

**Affiliations:** 1 Computer Science and Centre for Discrete Mathematics and its Applications (DIMAP), University of Warwick, Coventry, CV4 7AL UK; 2 Computer Science, Durham University, Durham, DH1 3LE UK; 3 Computer Science, Weizmann Institute, Rehovot, 7610001 Israel

**Keywords:** Component stability, Massively parallel computation, Lower bounds

## Abstract

In this paper, we study the power and limitations of component-stable algorithms in the low-space model of *massively parallel computation (*MPC*)*. Recently Ghaffari, Kuhn and Uitto (FOCS 2019) introduced the class of *component-stable* low-space MPC algorithms, which are, informally, those algorithms for which the outputs reported by the nodes in different connected components are required to be independent. This very natural notion was introduced to capture most (if not all) of the known efficient MPC algorithms to date, and it was the first general class of MPC algorithms for which one can show non-trivial conditional lower bounds. In this paper we enhance the framework of component-stable algorithms and investigate its effect on the complexity of randomized and deterministic low-space MPC. Our key contributions include: 1. We revise and formalize the lifting approach of Ghaffari, Kuhn and Uitto. This requires a very delicate amendment of the notion of component stability, which allows us to fill in gaps in the earlier arguments. 2. We also extend the framework to obtain conditional lower bounds for deterministic algorithms and fine-grained lower bounds that depend on the maximum degree $\Delta$. 3. We demonstrate a collection of natural graph problems for which deterministic component-unstable algorithms break the conditional lower bound obtained for component-stable algorithms. This implies that, in the context of deterministic algorithms, component-stable algorithms are conditionally weaker than the component-unstable ones. 4. We also show that the restriction to component-stable algorithms has an impact in the randomized setting. We present a natural problem which can be solved in *O*(1) rounds by a component-unstable MPC algorithm, but requires $\Omega (\log \log ^* n)$ rounds for any component-stable algorithm, conditioned on the connectivity conjecture. Altogether our results imply that component-stability might limit the computational power of the low-space MPC model, at least in certain contexts, paving the way for improved upper bounds that escape the conditional lower bound setting of Ghaffari, Kuhn, and Uitto.

## Introduction

The central goal of this paper is to advance our understanding of the computational power of low-space algorithms in the *massively parallel computation (*MPC*)* model. Our main focus is on the notion of *component-stable* low-space MPC algorithms introduced recently by Ghaffari, Kuhn and Uitto [26] as the first general class of MPC algorithms for which non-trivial conditional lower bounds can be obtained. Roughly speaking, in this class of algorithms the output of nodes in different connected components are required to be independent. While this definition has been introduced to capture most (if not all) of the known MPC algorithms to date, and the notion of component-stable algorithms seems quite natural and unlimited, we demonstrate its effect on the complexity of randomized and deterministic low-space MPC. Our first finding is that the notion of component-stability as defined in [26] is rather fragile and needs to be studied with care, leading us to a revision of this framework to make it robust. Our amended framework of component-stable algorithms allows us to fill in gaps in the earlier arguments and make it more applicable. In particular, the revised setup enables us to extend the framework of (conditional) lower bounds from [26] for component-stable randomized algorithms relating LOCAL algorithms and low-space MPC algorithms: we demonstrate that it can be parameterized with respect to the maximum graph degree $\Delta$ and holds also for deterministic algorithms, thereby making the framework more broadly applicable and proving for the first time a host of conditional lower bounds for a number of deterministic low-space component-stable MPC algorithms.

Next, we will show that for some natural problems there are low-space *component-unstable* MPC algorithms (both randomized and deterministic) that are significantly more powerful than their component-stable counterparts. So, rather than being a technical triviality, component-stability is in fact a significant restriction on the power of the low-space MPC model.

### Background

The rapid growth of massively parallel computation frameworks, such as MapReduce [18], Hadoop [47], Dryad [30], or Spark [49] resulted in the need of active research for understanding the computational power of such systems. The *massively parallel computation (*MPC*)* model, first introduced by Karloff et al. [31] (and later refined in [2, 7, 29]) has become the standard theoretical model of algorithmic study, as it provides a clean abstraction of these frameworks. Over the past years, this model has been receiving a major amount of interest by several independent communities in theory and beyond. In comparison to the classical PRAM model, the MPC model allows for a lot of local computation (in principle, unbounded) and enabled it to capture a more “coarse–grained” and meaningful aspect of parallelism (see, e.g., [3, 8, 17, 23, 28]).

In the MPC model, there are *M* machines and each of them has *S* words of local space at its disposal. Initially, each machine receives its share of the input.

In the context of graph problems where the input is a collection *V* of nodes and *E* of edges, $\vert V\vert = n$, $\vert E\vert = m$, the input is arbitrarily distributed among the machines (and so $S \cdot M \ge n + m$). In this model, the computation proceeds in synchronous *rounds* in which each machine processes its local data and performs an arbitrary local computation on its data. At the end of each round, machines exchange messages. Each message is sent only to a single machine specified by the machine that is sending the message. All messages sent and received by each machine in each round, as well as the output have to fit into the machine’s local space *S*.

Our focus in this paper is on the *low-space* setting where the local space of each machine is *strongly sublinear* in the number of nodes, i.e., $S = n^{\phi }$ for some $\phi \in (0,1)$. Our lower bounds will be against algorithms with any polynomial number of machines (i.e., $M = {\text {poly}}(n)$), while our upper bounds (with the exception of the non-uniform general derandomization of Lemma 32) will use at most $O(m+n^{1+\phi })$ global space (i.e., $M = O(\frac{m}{n^\phi } + n)$).

The low-space regime is particularly challenging due to the fact that a node’s edges cannot necessarily be stored on a single machine, but rather are scattered over several machines. Nevertheless, for many classical graph problems, ${\text {poly}}(\log n)$-round algorithms can be obtained, and recently we have also seen even *sublogarithmic* solutions. Ghaffari and Uitto [28] (see also [41]) presented a randomized graph sparsification technique resulting in $O(\sqrt{\log \Delta } \log \log \Delta + \log \log \log n)$-round algorithms for maximal matching and maxinmal independent set (MIS), where $\Delta$ is the maximum degree. This should be compared, for example, with maximal matching algorithms with significantly more local space: Lattanzi et al. [36] presented an $O(1/\varepsilon )$-round randomized algorithm using $O(n^{1+\varepsilon })$ local space and Behnezhad et al. [8] gave an $O(\log \log n)$-round randomized algorithm using *O*(*n*) local space (see also [4, 17, 23]). For the problem of $(\Delta +1)$-vertex coloring Chang et al. [10] showed a randomized low-space MPC algorithm that works in $O(\log \log \log n)$ rounds, when combined with the network decomposition result of Rohzoň and Ghaffari [45].

While we have seen some major advances in the design of low-space MPC algorithms, no (unconditional) hardness results are known for any of the above problems in the low-space MPC setting. A seminal work by Roughgarden et al. [44] provides an explanation for the lack of such lower bound results. They showed that obtaining any unconditional lower bound in the low-space MPC (for algorithms with an arbitrarily polynomial number of machines) setting ultimately leads to a breakthrough result in circuit complexity, namely that $\textsf {NC}^1 \subsetneqq \textsf {P}$. This work has opened up a new avenue towards proving *conditional* hardness results that are based on the widely believed *connectivity conjecture*. This conjecture (extensively used in our current paper) states that there is no $o(\log n)$-round (randomized) low-space MPC algorithm (even using any polynomial global space) for distinguishing between the input graph *G* being an *n*-length cycle and two $\frac{n}{2}$-length cycles. Several *global*^1^ problems were shown to be hard in low-space MPC under the conjecture [39, 48], but its relation to *local* problems was less clear.

The first conditional lower bounds for local problems in the low-space MPC setting were presented by a recent insightful paper of Ghaffari et al. [26]. This work provides a collection of conditional hardness results for classical local problems by drawing a new connection between the round complexity of a given problem in the LOCAL model [37], and its corresponding complexity in the low-space MPC model. Unlike the low-space MPC setting, for the LOCAL model, arguably one of the most extensively studied model in distributed computing, there is a rich collection of (unconditional) lower bound results. To enjoy these LOCAL lower bound results in our context, [26] presented a quite general technique that for many graph problems translates an $\Omega (r)$-round LOCAL lower bound (with an additional requirement of using shared randomness) into an $\Omega (\log r)$-round lower bound in the low-space MPC model *conditioned on the connectivity conjecture*. This beautiful lifting argument is surprisingly quite general, capturing the classical lower bounds for problems like MIS, maximal matching [34], LLL (Lovász Local Lemma), and sinkless orientation [9]. For example, one very strong implication of their technique is that conditioned on the connectivity conjecture, it shows that there is no randomized low-space MPC algorithm for (even approximate) maximal matching or MIS using $o(\log \log n)$ rounds.

The framework of Ghaffari et al. [26] has one main caveat, which at first glance appears to be quite negligible, a mere technicality. Their conditional lower bounds do not hold for *any* algorithms but rather only for the special class of *component-stable* MPC algorithms. The key property of these algorithms is that the output of the nodes in one connected component is independent of other components. More formally, in component-stable algorithms, the output of each node *v* is allowed to depend (deterministically) only on the node *v* itself, the initial distribution, the ID assignment of the connected component of *v*, and on the shared randomness. The class of component-stable algorithms is indeed quite natural, and at least by the time of publication of [26], it appeared to capture most, if not all, of the existing MPC algorithms, as explicitly noted by the authors:
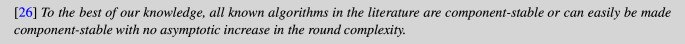


In this view, it appeared that the restriction to component-stable algorithms is no more than a minor technicality rather than an actual limitation on the low-space MPC model.

The first indication that component-stability might actually matter was provided by recent works [14, 16], which present deterministic low-space *component-unstable* MPC algorithms for several classic graph problems, even though the validity of solutions to these problems depends only on local information. Specifically, by derandomizing a basic graph sparsification technique, one can obtain $O(\log \Delta +\log \log n)$-round deterministic low-space component-unstable MPC algorithms for MIS, maximal matching, and $(\Delta +1)$-coloring. Czumaj et al. [15] later provided a more specialized derandomized algorithm improving the running time for $(\Delta +1)$-coloring to $O(\log \log \log n)$. A key ingredient of these algorithms is a global agreement on a logarithmic length seed, to be used by all nodes in order to simulate their randomized decisions. This global seed selection involves coordination between all the nodes, regardless of their components, thus yielding component-unstable algorithms. The component-instability here seems to be quite inherent to the derandomization technique, and it is unclear whether any component-stable method could perform equally well.

### Our aims

In this paper *we thoroughly investigate the concept of component-stability and its impact on randomized and deterministic low-space* MPC * algorithms*.

Upon examining the notion of component-stability in detail and after attempts to broaden its applications, it becomes apparent that the concept is highly sensitive to the exact definition used, and that one must be very careful in specifying what information the outputs of component-stable algorithms may depend on. For example, we must precisely specify whether we allow component-stable algorithms’ outputs to depend on the input size *n*, and we find that either choice here holds problematic implications for the current lower bounds and for the analysis due to Ghaffari et al. [26].

This raises the first main question of our work:
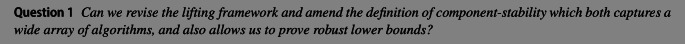


Having fixed such a definition, we ask to what extent component-stability restricts MPC algorithms, and whether the concept is indeed a technicality or actually a significant limitation. The lifting arguments of [26] are designed for randomized algorithms, which raises the following question:
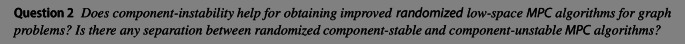


We then turn to consider the impact of component-stability on *deterministic* low-space MPC algorithms. Since the recent derandomization technique of [14, 16] leads to inherently component-unstable algorithms, we ask:
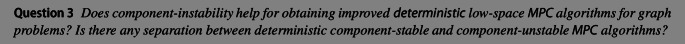


Understanding the gap between randomized and deterministic solutions is one of the most fundamental and long-standing questions in graph algorithms. In a very related context, the last few years provided a sequences of major breakthrough results which almost tightly characterize the gap between the deterministic and randomized complexities for *distributed computation* (in the LOCAL model). Rohzoň and Ghaffari [45] settled a several-decades-old open problems by presenting a deterministic polylogarithmic algorithm for network decomposition. Their result implies that any polylogarithmic-time randomized algorithm for LCL problems [12, 40] can be derandomized to a polylogarithmic-time deterministic algorithm. In other words, *randomization does not help in the polylogarithmic-time regime*. On the other hand, Chang et al.  [12] showed that *in the sub-logarithmic-time regime, randomization might provide an exponential benefit*. For example, for $\Delta$-coloring of trees of maximum degree $\Delta$ there are $\Theta (\log _{\Delta }\log n)$ randomized-round algorithms and a deterministic lower bound of $\Omega (\log _{\Delta } n)$ rounds. Balliu et al. [5] recently showed that maximal matching and maximal independent sets cannot be found by $o(\Delta + \log \log n/ \log \log \log n)$-round randomized algorithms, and deterministically in $o(\Delta + \log n)$ rounds, thus providing an exponential gap in the lower bound for bounded-degree graphs. Finally, Chang et al. [11] presented additional separation results for edge-coloring: $(2\Delta -2)$-edge-coloring requires $\Omega (\log _{\Delta } \log n)$ randomized rounds, and $\Omega (\log _\Delta n)$ deterministic rounds. In view of these separation results, we therefore ask in the context of related MPC computation:
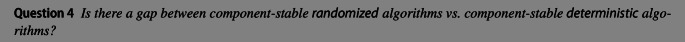


In this paper, we answer all four questions in the affirmative.

### Our contributions

In this paper, we study the power and limitations of component-stable algorithms in the low-space MPC model. This class of algorithms is to this date the only class for which conditional lower bound for local graph problems can be obtained. Our main contribution is in demonstrating the impact of this property on the complexity of randomized and deterministic local graph problems. Towards that goal, we informally define the following four complexity classes:

- *DetMPC* deterministic low-space component-unstable MPC algorithms,
- *S-DetMPC* deterministic low-space component-stable MPC algorithms,
- *RandMPC* randomized low-space component-unstable MPC algorithms,
- *S-RandMPC* randomized low-space component-stable MPC algorithms.

We refer to Sect. 2.5 for the precise definitions.

#### A robust lifting framework

We rectify the framework of low-space component-stable MPC algorithms due to Ghaffari et al. [26]. We study the framework in detail and demonstrate that in order to be broadly applicable, many aspects of the original setting are highly sensitive to the exact definition used, and require amendments to carefully specify what information the outputs of component-stable algorithms may depend on. We present a modified framework, reaching a revised definition of component-stability (see Definition 5) which both encompasses many existing MPC algorithms, and for which robust conditional lower bounds can be shown. This answers Question 1.

#### Extensions to deterministic and degree-dependent lower bounds

Our revised framework not only recovers all main results from the framework of Ghaffari et al. [26], but also extends the arguments to include conditional lower bounds for *deterministic algorithms* and fine-grained lower bounds that depend on $\Delta$. While our main theorem (Theorem 5) lifting LOCAL lower bounds to component-stable MPC algorithms has several subtle assumptions, the main, informal claim is that for many graph problems ${\mathcal {P}}$, if ${\mathcal {P}}$ has a $T(n,\Delta )$-round (randomized or deterministic) lower bound in the LOCAL model, then assuming the connectivity conjecture, any low-space component-stable (respectively, randomized or deterministic) MPC algorithm solving ${\mathcal {P}}$ requires $\Omega (\log T(n,\Delta ))$ rounds.

#### Instability helps randomized MPC algorithms

To address Question 2, we consider the problem of finding a large (specifically, of size $\Omega (n/\Delta )$) independent set. This problem has been recently studied by Kawarabayashi et al. [32], who provided a randomized LOCAL lower bound of $\Omega (\log ^* n)$ rounds (for a specific range of $\Delta$). We show that their lower bound can be adapted to our revised lower-bound lifting framework of Theorem 5, obtaining a conditional lower bound of $\Omega (\log \log ^* n)$ rounds for component-stable MPC algorithms.^2^ In contrast, we present a very simple *O*(1)-round component-unstable randomized algorithm for the problem. In fact, this algorithm can further be *derandomized* within *O*(1) rounds (see Theorem 30), demonstrating an instance in which deterministic component-unstable algorithms are more powerful even than randomized component-stable algorithms (i.e., $\textsf {DetMPC} \nsubseteq \textsf {S-RandMPC}$).

##### Theorem 1

Conditioned on the connectivity conjecture, any component-stable low-space MPC algorithm for computing an independent set of size $\Omega (n/\Delta )$ on *n*-node graphs (for the full range of $\Delta \in [1,n)$) and succeeding with probability at least $1-\frac{1}{n}$, requires $\Omega (\log \log ^*n)$ rounds. This problem admits a simple *O*(1)-round randomized low-space MPC algorithm which is component-unstable; additionally, the algorithm can be derandomized within *O*(1) rounds.

Stated in complexity language, Theorem 1 provides a *separation between the class of* RandMPC * and* S-RandMPC (conditioned on the connectivity conjecture, see Theorem 7). The basic observation providing this separation is the fact that one can easily compute in *O*(1) rounds (even in the LOCAL model) an independent set of $\Omega (n/\Delta )$ nodes *in expectation*. In the LOCAL model, we need provably longer to achieve a high-probability success guarantee of $1-\frac{1}{n}$. In the low-space MPC model, however, we can perform the process of *success probabiliy amplification*: we run $\Theta (\log n)$ *parallel* repetitions of the basic algorithm, and choose a successful one if such exists, amplifying the success probability to $1-\frac{1}{n}$ without any slow-down. This powerful process, though, is inherently component-unstable, since it relies on globally agreeing on one of the repetitions to use.^3^

#### Instability helps deterministic MPC algorithms

We then turn to consider the effect of component-stability on deterministic MPC algorithms. While the original setup of Ghaffari et al. [26] had been designed only for randomized algorithms, the revised framework developed in our paper in Sect. 2.4.6 extends naturally to the deterministic setting, providing a robust deterministic lifting analog in Theorem 5. Theorem 5 provides a general framework lifting unconditional deterministic lower bounds for the LOCAL model for many natural graph problems to conditional lower bounds for low-space component-stable MPC algorithms in the same way as the randomized framework in [26].

We then turn to show that with component-instability one can in fact surpass these conditional lower bounds and present several results showing a *separation between* DetMPC * and* S-DetMPC (conditioned on the connectivity conjecture) and positively answering Question 3. In Sect. 4.2, we show that for several problems closely related to LLL, including sinkless orientation and some variants of edge-coloring and vertex-coloring, component-instability helps for deterministic algorithms. Finally, in Sect. 4.3, we demonstrate a similar result for the class of all LOCAL *extendable* algorithms by combining the lifting of deterministic LOCAL lower bounds in Theorem 5 with a derandomization technique using pseudorandom generators. To demonstrate the applicability of this derandomization recipe, we show how it can be used to *improve the deterministic running times* of two cornerstone problems in low-space MPC: *maximal independent set* and *maximal matching*. And so, on one hand we prove (Theorem 26) that conditioned on the connectivity conjecture, there is no deterministic low-space component-stable MPC algorithm that computes a maximal matching or maximal independent set, even in forests, in $o(\log \Delta +\log \log n)$ rounds, and on the other hand, we give a deterministic low-space component-unstable MPC algorithm for these problem running in $O(\log \log \Delta + \log \log \log n)$ rounds (when $\Delta =2^{\log ^{o(1)} n}$, Theorem 25). (The resulting MPC algorithm must either perform heavy local computations, or alternatively, the underlying PRGs can be hard-coded in the MPC machines for a non-uniform but computationally-efficient algorithm.)

#### Relations between randomized and deterministic MPC algorithms

Finally, we consider the interesting gap between randomized and deterministic algorithms in the low-space MPC setting. As observed by [12, 25] (and see Lemma 31), randomized algorithms that succeed with probability of $1-1/2^{n^2}$ can be turned into non-uniform deterministic algorithms. This result can also be extended to the low-space MPC setting, with some caveat. In contrast to the LOCAL model where the space of the nodes is unlimited, in the low-space MPC setting, the transformation implied by [12, 25] yields a non-explicit algorithm (see Lemma 31). By using success probability amplification with ${\text {poly}}(n)$ machines, one can boost the success guarantee of any randomized MPC algorithm from $1-1/{\text {poly}}(n)$ to $1-1/2^{n^2}$ without any slowdown in the round complexity. From the complexity perspective, restricting to non-uniform and non-explicit computation, one therefore finds that $\textsf {DetMPC} = \textsf {RandMPC}$, see Theorem 9. For some specific problems, we can perform more careful derandomization methods that *do not* cause the resulting deterministic algorithms to be non-uniform, non-explicit, or to use excessive global space, demonstrating that component-stability restricts power even without these allowances.

Turning our focus to low-space component-stable MPC algorithms, here we can provide a conditional separation between randomized and deterministic algorithms (Theorem 13), positively answering Question 4. This separation follows by combining (i) the conditional lifting for randomized component-stable algorithms and deterministic component-stable algorithm with (ii) local problems for which there is provable gap in their randomized and deterministic LOCAL complexity.

#### Complexity summary

Let us summarize the complexity results, assuming the connectivity conjecture, and allowing non-uniform MPC algorithms. Our study demonstrates that in low-space MPC, component-unstable algorithms are provably stronger than their component-stable counterparts, both for deterministic and randomized algorithms (Theorems 6, 7). Further, for component-stable algorithms, randomized algorithms are provably stronger than their deterministic counterparts (Theorem 8). However, for arbitrary (possibly component-unstable) algorithms this is not the case: any randomized algorithm can be efficiently simulated by a deterministic one (Lemma 32 and Theorem 9).

## Revised framework of component stability

In this section we suggest an array of changes that may be made to the framework of component-stable algorithms due to Ghaffari et al. [26], in order to reach a revised definition of component-stability (Definition 5) which both encompasses many existing randomized MPC algorithms, and for which robust conditional lower bounds can be shown. These changes also allow us to extend the original setting to both deterministic algorithms and those that have running-time dependency on maximum degree $\Delta$.

### Discussion of definitions of component stability

Let us first present the description of component stability from [26, Section II]:
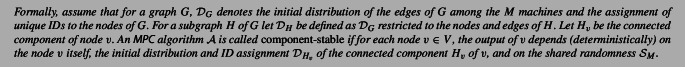


We informally sketch the line of argument of [26] that leverages this definition to lift LOCAL lower bounds to MPC: first, it is shown that if there is a LOCAL lower bound for a problem *P*, and an MPC algorithm $A_{MPC}$ is able to solve *P* faster than the $\log$ of the LOCAL lower bound, there there must exist two graphs *G* and $G'$, which are locally indistinguishable but on which $A_{MPC}$’s output must differ (at least with some sufficiently large probability). In the terminology of [26], $A_{MPC}$ must be ‘farsighted’: able to somehow make use of information from far-away nodes.

These graphs *G* and $G'$, and the assumed algorithm $A_{MPC}$, are then ingeniously used to construct an algorithm $B_{st-conn}$ that solves a connectivity problem conjectured to be hard. Specifically, $B_{st-conn}$ constructs a pair of simulation graphs based on its input to the connectivity problem. These simulation graphs consist of many disjoint copies of induced subgraphs of *G* and $G'$ respectively. The construction is made in such a way that a *full* copy of *G* and $G'$ only appears if two particular nodes (designated *s* and *t*) are connected in the input graph for the connectivity problem.

$B_{st-conn}$ simulates $A_{MPC}$ on this pair of simulation graphs. If *s* and *t* are connected, then full copies of *G* and $G'$ are present as connected components in the simulation graphs, and $A_{MPC}$ should return *different* outputs on them with sufficiently high probability. Otherwise, there are no full copies of *G* and $G'$, and $A_{MPC}$ returns the same outputs on both simulation graphs. This difference in behavior is exploited to allow $B_{st-conn}$ to determine whether *s* and *t* are connected, and solve the connectivity problem faster than is conjectured to be possible.

The property of component-stability is crucial in this last step: we require that $A_{MPC}$ behaves the same on *G* and $G'$ when they are connected components of the (much larger) simulation graphs as it does when they are the entire input (as was the case when showing that $A_{MPC}$ was farsighted). Otherwise, we could not say anything about $A_{MPC}$’s output on the simulation graphs. It transpires that this argument is quite fragile, and highly sensitive to the precise definition of component-stability used. We discuss some of the issues below.

#### Randomized component-stable algorithms must be allowed dependency on *n*

The first major point of consideration is that, as defined in [26], the output of a node *v* under a component-stable algorithm must depend only on shared randomness, the IDs of *v* and its component, and the input distribution of edges to machines. In particular, no provision is made for dependency on the number of nodes *n* in the input graph, and indeed, the arguments of [26] seem to forbid it,^4^ This is somewhat counter-intuitive for MPC algorithms: while a LOCAL algorithm can never determine *n* unless it is given as input (and therefore it is commonly assumed that we provide the algorithm with at least a polynomial estimate of *n*), an MPC algorithm can easily do so in *O*(1) rounds, by simply summing counts of the number of nodes held on each machine.^5^ We can therefore assume any such algorithm *has* knowledge of the exact value of *n*, and natural algorithmic approaches would generally make use of this knowledge.

Furthermore, the *success* probability of correct randomized algorithms is defined to be at least $1-\frac{1}{n}$, in accordance with the standard definition of *with high probability correctness*. This causes a contradiction for algorithms with no output dependency on *n*:

Consider a correct component-stable MPC algorithm *A* for a problem in which the validity of a node’s output can be verified by seeing its connected component (we will formalize this notion later), running on a *n*-node graph. This algorithm must produce a valid output for each node in the graph with probability at least $1-\frac{1}{n}$.

We now add $\eta$ disconnected nodes to the input graph. If *A*’s output does not have any dependency on *n*, then it must be unchanged at each of the original nodes, since they are in an unchanged connected component. However, *A* must succeed on the new graph with probability at least $1-\frac{1}{n+\eta }$. Since *A* is component-stable, the probability that it succeeds on all of the nodes of the original graph is at least as high as the probability that it succeeds on the whole graph, i.e., $1-\frac{1}{n+\eta }$. So, *A* must succeed on the original graph with probability at least $1-\frac{1}{n+\eta }$, and since we can set $\eta$ arbitrarily high, must therefore succeed with certainty.

In short, a definition of component-stability which does not allow any dependency on *n* essentially rules out all randomized algorithms with probabilistic success guarantees (though does still permit Las Vegas randomized algorithms), and so does not capture most of the problems to which the framework was originally applied.

#### If we allow dependency on *n*, we must restrict the class of problems in order to obtain MPC lower bounds

We have seen that, to give results which apply to probabilistic algorithms, we must allow dependency on *n*. However, we cannot then hope to obtain a result akin to Theorem I.4 of [26] for *all* graph problems.

As an example, consider the following problem: each node must output **YES** if the entire graph is a simple path with consecutive node IDs, and **NO** otherwise. Note that there is only one possible correct output for each node *v*, and that this output is a deterministic function of its component and the value of *n* (since *v*’s output should be **YES** iff its component is an *n*-node path with consecutive IDs). Furthermore, there is an *O*(1)-round MPC algorithm for the problem: it is straightforward to check whether there are two nodes of degree 1, $n-2$ nodes of degree 2, and that each node’s 1-hop neighborhood is consistent with being in a path of consecutive IDs. So, if component-stability is defined to allow dependency on *n*, an *O*(1)-round deterministic component-stable algorithm for the problem exists.

However, the problem has a trivial $n-1$-round (randomized) LOCAL lower bound, since a **YES** instance can be changed to a **NO** instance by only altering the ID of one endpoint of the path, and the other endpoint requires $n-1$ rounds to detect this change. Hence, we cannot hope for a universal method of lifting LOCAL lower bounds to non-trivial component-stable MPC lower bounds if component-stability allows dependency on *n*.

We will see, though, that such counterexamples are necessarily quite contrived, and that we *can* prove such a result for a class that includes most problems of interest (such as, e.g., *all locally-checkable (*LCL*)* problems, see Sect. 2.3).

#### Uniqueness of identifiers

It is common in both LOCAL and MPC to assume that nodes of the input graph are equipped with identifiers (IDs) that are unique throughout the entire graph. This assumption, however, is somewhat at odds with the concept of component-stability: if, for example, a disconnected node is added to a valid graph, sharing an ID with an existing node, then the input becomes invalid. So, outputs for the original nodes are now allowed to change arbitrarily, even though their components have not altered.

We could instead require that IDs are only component-unique (i.e., they are allowed to be shared by disconnected nodes, but not by connected ones). This is a weaker assumption which aligns well with component-stability, and is still sufficient for LOCAL algorithms (in which nodes have no interaction with or dependency on disconnected nodes).

This approach, though, presents a problem in MPC. Unlike in LOCAL, where nodes are inherently separate computational entities which only need IDs for symmetry-breaking (particularly for deterministic or shared randomness algorithms), in MPC an input graph node essentially *is* its ID. The input is given only as a binary encoding of the IDs of nodes and edges, and so any two nodes with the same ID will be contracted to a single node when this input is interpreted as a graph. As a consequence, MPC algorithms cannot work with graphs in which IDs are only component-unique.

Our solution to this problem is to separate the two functions of IDs. We will assume that IDs are only component-unique, and that component-stable MPC algorithms can depend on these. However, we also provide MPC algorithms with fully-unique *names* for nodes, whose purpose is *only* to allow the algorithm to distinguish the input graph’s nodes as separate objects. Accordingly, we do not allow the output of component-stable algorithms to depend on the names.^6^

#### Initial distribution of input

The definition of [26] allows MPC algorithms’ outputs to depend on the initial distribution of the input to the machines. While this is natural to do, we observe that under our definition it is not necessary: given a component-stable algorithm $A_{MPC}$ whose output depends on this distribution, we can always create a new component-stable $B_{MPC}$ which does not.

Specifically, since the nodes have unique *poly*(*n*) names (and we can also give edges unique *poly*(*n*) names based on their endpoints), and we are allowed any *poly*(*n*) number of machines, algorithm $B_{MPC}$ can first (in one round) redistribute each node and edge of the input to its own dedicated machine, with the same name as the corresponding node or edge. Then, it simulates $A_{MPC}$, and reaches a valid output, which is now independent of the initial distribution. Since $A_{MPC}$’s output is component-stable, $B_{MPC}$’s is also.^7^

Therefore, a lower bound for component-stable algorithms that depend on input distribution implies a lower bound for those that do not. So, we can disallow this dependency from the definition without weakening our results.

### Graph families

In this section, we make some definitions concerning the input graphs on which MPC algorithms run. Firstly, to address the problem concerning uniqueness of identifiers, we define *legal graphs* to be those with separate unique node names and component-unique node IDs as discussed:

#### Definition 1

A graph *G* is called **legal** if it is equipped with functions $\textsf {ID}, \textsf {name}: V(G)\rightarrow [poly(n)]$ providing nodes with IDs and names, such that all names are fully unique and all IDs are unique in every connected component.

Throughout the paper, we will always assume that input graphs for MPC are legal (and we will *ensure it when constructing inputs ourselves*). For component-stable algorithms, this is to allow a weaker dependency on the IDs and not the names, as discussed above. For component-unstable algorithms, it is no different from the standard (fully-unique IDs) requirement, since their outputs are allowed to depend on the names, and so we can simply use the names as IDs.

Next, we make a definition which will allow us to show MPC lower bounds on specific families of graphs. LOCAL lower bounds are often proven using graphs from some specific family ${\mathcal {H}}$ as the *“hard instances”*: in particular, many such bounds are proven on trees. Lower bounds on restricted families of graphs are stronger than those on the class of all graphs, and can also provide more meaningful hardness results for problems which are only *possible* on restricted families (such as $\Delta$-vertex coloring, see Theorem 22). When lifting LOCAL lower bounds on restricted graph families to MPC, we therefore wish to preserve the family on which the lower bound holds.

As discussed in Sect. 2.1, the lines of argument made both by [26] and this work involve construction of a *simulation graph* *H* as the “hard instance” in MPC. These simulation graphs (from the proof of Lemma IV.2 of [26] and Lemma 11 here) are constructed differently, but both consist of disjoint unions of induced subgraphs of some hard instance *G* for the LOCAL lower bound.

The simulation graph *H* is not necessarily in ${\mathcal {H}}$, if ${\mathcal {H}}$ is an arbitrary graph family, and is not therefore a valid input for an MPC algorithm defined to run on ${\mathcal {H}}$. This is not necessarily a problem for [26], since one could require that their definition of component-stability should hold *even if algorithms are given an invalid input*. Under this definition, the output of a component-stable MPC algorithm $A_{MPC}$ on *H* must be the disjoint union of $A_{MPC}$’s outputs on its connected components (which, in this case, are in $\mathcal H$) separately, and this circumvents the need for *H* itself to be in ${\mathcal {H}}$.

However, as we notice in Sect. 2.1, to incorporate non-trivial randomized algorithms, any suitable definition of component-stable algorithms must allow dependency on *n*. Then, the output of $A_{MPC}$ of *H* need not be the union of that on *H*’s connected components, since the inputs have differing values of *n*. This necessitates several changes from the proofs of [26], one of which is that we *do* require $H\in {\mathcal {H}}$.

To ensure that this is the case, we prove our lower-bound lifting argument only for the following families of graphs:

#### Definition 2

(*Normal families of graphs*) A class of graphs ${\mathcal {H}}$ is called **normal** if it is hereditary (i.e., closed under removal of nodes) and closed under disjoint union.

The set of *all graphs* is a normal family, and can always be used in the worst case. Further, observe that the *class of all trees is not a normal family* of graphs; however, the family of *all forests* is normal. Therefore, for example, Theorem 5 implies that LOCAL lower bounds on *trees* can be lifted to conditional MPC lower bounds on *forests* (but not trees).

### Types of graph problems and replicability

We next define the types of *problems* we will encompass with this work. We will focus on graph problems, and let ${\mathbb {G}}$ be the collection of all legal input graph instances.

We consider only graph problems where *each node of the input graph must output some label* from a finite set $\Sigma$. For example, for the vertex coloring problem the label of a node corresponds to its color, and for the independent set problem, the label corresponds to the indicator variable whether the node is in the independent set returned.

As in [26], we do not explicitly output labels for edges. However, to apply our results to problems in which only edges are given output labels (such as matching, edge coloring, or sinkless orientation), we can simply redefine the problem as vertex-labeling on the *line graph* (where the vertices represent edges of the input graph, with IDs and names given by Cartesian products of the IDs and names of their endpoints). For any normal graph class ${\mathcal {H}}$, the family of $\mathcal {L_H}$ of line graphs of graphs from ${\mathcal {H}}$ is also normal. Working on the line graph increases number of nodes *n* at most quadratically and maximum degree $\Delta$ at most by a factor of 2, and in LOCAL requires only 1 extra round. We will see that performing this conversion to the line graph will allow us to obtain results for edge-labeling problems without any asymptotic change in bounds. We can then convert back to recover a solution to the problem on the original graph.

A graph problem is then defined by a collection of *valid* outputs for each possible pair (topology, IDs) of a legal input graph. Importantly, we do *not* allow validity to be dependent on the *names* of graph nodes (though these names are part of any legal input). That is, given a particular input graph topology and set of IDs, the collection of valid outputs must be consistent regardless of node names. This is because component-stable outputs are not allowed to depend on names, so most problems which allowed solution-dependency on names would be trivially unsolvable by component-stable algorithms. In any case, names were introduced solely to allow MPC algorithms to distinguish nodes as objects, and should not be considered part of problems.

The goal of any algorithm for the problem is then to provide a valid output for the specific legal input it was given. For many problems it is useful to have a concept of the output of a *particular node* being valid. The overall output is then valid if all nodes’ outputs are valid. To capture this concept, we define the following sub-class of problems:

#### Definition 3

For $r \in {\mathbb {N}}$, an *r***-radius checkable problem** is defined by a collection of valid outputs for each *r*-radius centered graph equipped with unique IDs.^8^ The output of a node *v* in input graph *G* is deemed valid if the centered graph given by its *r*-radius ball, and the outputs thereof, is a member of this valid collection. An overall output on *G* is valid if all nodes’ outputs are valid.

An *r-radius centered graph* here is simply a connected graph with a designated center node, from which all other nodes are of distance at most *r*.

One can see that *r*-radius checkable problems are precisely those whose solutions can be verified in *r* rounds of LOCAL. Note that the majority of graph problems of interest are *r*-radius-checkable for some $r\le n$: for example, the vertex coloring problem requires that each node outputs a color distinct from the colors output by its neighboring nodes, and thus is easily 1-radius-checkable. Similarly, all LCL (*locally-checkable labeling*, see, e.g., [12, 40]) problems, a commonly studied class particularly from a lower-bounds perspective, are *O*(1)-radius checkable. Still, some natural problems are not *n*-radius checkable problems: most notably, approximation problems are not, since there is no notion of a node’s validity, and nor can nodes determine overall validity by seeing their *n*-radius ball (i.e., their entire connected component). So, while some of our results concern *r*-radius checkable problems (such as those in Sect. 4.3), our main lower bounds results will use a more general class of problems, see below, in order to incorporate approximation problems.

#### Replicable graph problems

We have seen, from Sect. 2.1, that to transfer LOCAL lower bounds to MPC, under a definition of component-stability that includes randomized algorithms (and so allows dependency on *n*), one must restrict the class of problems, since some (contrived) problems have $\Omega (n)$-round LOCAL lower bounds and *O*(1)-round MPC algorithms. Our goal in this section is to make the minimal restriction needed to facilitate such lower-bound lifting arguments.

During proof of Theorem 5 (our main lower-bound lifting theorem), we will consider multiple disjoint copies of the input graph enhanced by isolated nodes. To facilitate this concept in our analysis, we introduce the notion of *replicable graph problems*.

##### Definition 4

A graph problem is *R*-**replicable** if it satisfies the following property. For any

- graph $$G \in {\mathbb {G}}$$ with $$\vert V(G)\vert \ge 2$$,
- output labeling $$L: V(G) \rightarrow \Sigma $$,
- individual output label $\ell \in \Sigma$, and
- graph $\Gamma _G$ which is a disjoint union of at least $\vert V(G)\vert ^R$ disjoint copies of *G* (with the same IDs as *G*) and fewer than $\vert V(G)\vert$ isolated nodes (with the same ID as each other),

let output labeling $L'$ on $\Gamma _G$ be given by *L* on each copy of *G*, and $\ell$ on each isolated node. Then, if $L'$ is valid on $\Gamma _G$, *L* must be valid on *G*.

Note that replicability is monotonic in *R*, i.e., if *P* is *R*-replicable then *P* is also $(R+1)$-replicable, since any $\Gamma _G$ satisfying the construction of $(R+1)$-replicability also satisfies the construction for *R*-replicability.

The definition of replicability may seem unnatural: it is designed to align with a specific construction needed in proof of Lemma 10 (and ultimately Theorem 5). However, we argue that the vast majority of natural graph problems are replicable. We first show all that *r*-radius-checkable problems (and hence all LCL problems [12, 40]) are replicable;

##### Lemma 2

Any *r*-radius-checkable problem is 0-replicable.

##### Proof

For any *r*-radius-checkable problem, the validity of the output of a connected component depends only on the IDs and topology of the connected component. Any $\Gamma _G$ satisfying the construction of 0-replicability contains at least one copy of *G* as a connected component. So, for the output on $L'$ on $\Gamma _G$ to be valid, the output *L* must be valid on *G*. $\square$

Further, a major strength of our framework is that *most approximation problems are also replicable*. As an example, we show replicability for the problem of finding an independent set of size $\Omega (n/\Delta )$ (which is an $\Omega (1/\Delta )$-approximation of the maximum independent set), a problem for which we will later (in Sect. 5) show a separation between component-stable and component-unstable algorithms.

##### Lemma 3

The problem of finding an independent set of size $\Omega (n/\Delta )$ (on graphs with $\Delta \ge 1$) is 2-replicable.

##### Proof

Let $c > 0$ be some fixed constant. Consider a graph *G* on *n* nodes, with maximum degree $\Delta$, and a graph $\Gamma _G$ consisting of $k \ge n^2$ copies of *G* and fewer than *n* isolated nodes. Note that $\Gamma _G$ has at least *kn* nodes. Assume we have some output valid labeling $L'$ on $\Gamma _G$, which corresponds to an independent set of size at least $ckn/\Delta$, and in which each copy of *G* is labeled identically, as is each isolated node. More than $ckn/\Delta -n$ of the nodes in the independent set must be in copies of *G* (since there are fewer than *n* nodes not in copies of *G*). Since each copy of *G* is labeled identically, each must contain more than

$$\begin{aligned} \frac{ckn/\Delta -n}{k}&\ge \frac{cn}{\Delta } - \frac{1}{n} \ge \frac{cn}{2\Delta } \end{aligned}$$

nodes in the independent set (for $n\ge 2/c$), and therefore the output on *G* is a valid $\Omega (n/\Delta )$-independent set. $\square$

Similarly, we have a related lemma for approximate matching, one of the central problems demonstrating the power of our framework summarized in Theorem 5 (which will yield also the conditional hardness of the approximate maximum matching problem on MPC). The same arguments can also straightforwardly show that $\Omega (1)$-approximation of maximum matching and minimum vertex cover are *O*(1)-replicable.

##### Lemma 4

The problem of finding an $\Omega (1)$-approximation of maximum matching is 2-replicable.

##### Proof

To fit maximal matching into our vertex-labeling definition for problems, we characterize it as maximal independent set on the (normal) family of *line graphs*, as discussed above. An $\Omega (1)$-approximation of maximum matching on an input graph corresponds to an $\Omega (1)$-approximate maximal independent set on its line graph. Let *G* be such a line graph, on *n* nodes, and let $c > 0$ be some fixed constant. We consider a graph $\Gamma _G$ consisting of $k \ge n^2$ copies of *G* and fewer than *n* isolated nodes. Again, $\Gamma _G$ has at least *kn* nodes, and we note that (denoting $\Lambda (H)$ to be the size of the MIS of a graph *H*) $\Lambda (\Gamma _G) \ge k\Lambda (G)$. Assume we have some output valid labeling $L'$ on $\Gamma _G$, which corresponds to an independent set of size at least $c \Lambda (\Gamma _G)$, and in which each copy of *G* is labeled identically, as is each isolated node. More than $c \Lambda (\Gamma _G)-n$ of the nodes in the independent set must be in copies of *G* (since there are fewer than *n* nodes not in copies of *G*). Since each of the *k* copies of *G* is labeled identically, each must contain more than

$$\begin{aligned} \frac{c \Lambda (\Gamma _G)-n}{k}&\ge \frac{ck\Lambda (G)}{k} - \frac{1}{n} \ge \frac{c\Lambda (G)}{2} \end{aligned}$$

nodes in the independent set (for $n\ge 2/c$), and therefore the output on *G* is a valid $\Omega (1)$-approximate MIS, corresponding to an $\Omega (1)$-approximate maximal matching in the input graph. $\square$

### Algorithm definitions, and revised definition of component-stability

Once we have defined *problems*, as considered in our paper, we may define LOCAL and MPC algorithms that solve them, and in particular, give a formal, amended definition of component-stable algorithms used in this paper, taking into account the discussion above.

#### LOCAL algorithms

Our formal definition of algorithms in the LOCAL model used in this paper is as follows:

***Input.***

LOCAL algorithms receive as input an *n*-node graph *G*, with component-unique IDs for each node. Randomized algorithms also provide each node with access to a *shared, unbounded, random seed* ${\mathcal {S}}$. Algorithms are provided with the exact value of the maximum degree $\Delta$, and an *input size estimate* *N* of *n* such that $n\le N \le poly(n)$.^9^

The nodes of the input graph are the computational entities, and each initially has knowledge of its adjacent edges in *G* (i.e., the IDs of their other endpoints). The computation proceeds in synchronous rounds, and in each round, a node may send an arbitrary message along each of its adjacent edges. At the termination of the algorithm, each node must give an output label from $\Sigma$.

#### Output

Correct deterministic algorithms must always provide a valid overall output labeling for the problem, on every input; randomized algorithms must give a valid labeling with probability at least $1-\frac{1}{N}$, over the distribution of the random seed ${\mathcal {S}}$, for any input.

#### Shared randomness

Given that MPC algorithms naturally allow shared randomness, it is important for our study of randomized LOCAL algorithms to allow the nodes to have access to shared randomness too. The use of *shared randomness* is non-standard in the LOCAL model, where one typically assumes only private randomness. However, as shown by Ghaffari et al. [26, Section V], many of the existing LOCAL lower bounds can be extended (and without any asymptotic loss in their LOCAL round complexity) to hold also if the nodes have access to shared randomness. (Notice that the notion of shared randomness is only relevant to randomized algorithms, and hence, for deterministic complexity one can use the existing deterministic LOCAL lower bounds without any constraints, as black box results.)

#### MPC algorithms

We use the standard definition of MPC algorithms (see, e.g., [3, 8, 17, 23, 28, 31]) amended to fit the framework of low-space MPC s used in the paper.

***Input.***

MPC algorithms receive as input a legal *n*-node graph *G*, distributed arbitrarily over *poly*(*n*) machines, each with local space $O(n^{\phi })$ for some $\phi <1$. Randomized algorithms also provide each node with access to a shared, random seed ${\mathcal {S}}$ of *poly*(*n*) bits (again distributed arbitrarily among machines). We do not assume that $\Delta$ or *n* are given explicitly as input, but MPC algorithms can determine them easily in *O*(1) rounds, so we may assume knowledge thereof.

Computation proceeds in synchronous rounds, and in each round, a machine first perform an arbitrary local computations on its local data and then may send and receive a total of $O(n^{\phi })$ information, divided between any other machines as desired. At the termination of the algorithm, each machine must give an output label from $\Sigma$ for each node it received in the initial distribution.

***Output.***

Correct deterministic algorithms must always provide a valid overall output labeling for the problem, on every output; randomized algorithms must give a valid labeling with probability at least $1 - \frac{1}{n}$, over the distribution of the random seed ${\mathcal {S}}$, for any input.

#### Computation in MPC algorithms

While we are mainly using the most standard setup of MPC algorithms, closely following, e.g., [3, 8, 17, 23, 28, 31], occasionally we will use some features which (while often standard) are less commonly used.

The standard MPC model assumes that in each synchronous rounds, each machine performs arbitrary local computations on its data (fitting its local memory of size $S = O(n^{\phi })$) and then the machines simultaneously exchange messages, in a way that each machine is sender and receiver of up to *O*(*S*) messages. While some papers also consider the sequential running time of any single MPC machine in every round, the main focus of our study is primarily on the information theoretic aspects of understanding the round complexity in MPC algorithms. (Notice that unbounded local computation assumption is standard in the classical distributed models as LOCAL, CONGEST, and CONGESTED CLIQUE.) As the result, while many of our algorithms perform only ${\text {poly}}(n)$-time computations, occasionally we will allow MPC machines to perform *heavy local computations*, up to $2^{O(S)}$ local computations in a round; still, the space used on a single machine remains $S = O(n^{\phi })$. Our results show that allowing such heavy computations might provide advantageous in the context of deterministic algorithms and derandomization, however they are not necessary to find examples of component-unstable deterministic algorithms which surpass component-stable conditional lower bounds.

Furthermore, while typically one is concerned with the design of uniform MPC algorithms, as it has been observed by Fish et al. [20], the original setup of MPC (e.g., [31]) leads naturally to the non-uniform model of computation. Most of the MPC algorithms presented in our paper are uniform, but occasionally we use *non-uniform algorithms*. In our setting, this means that the MPC algorithm, on each single machine initially knows the number of nodes *n* (or its estimation), and possibly different algorithms are used for different values of *n*. This can be also seen as having some non-uniform advice hardwired in the algorithms on individual MPC machines (or as Boolean circuits; for more details, see, e.g., Section 7.1.1 in [46]).

Finally, some of the non-uniform MPC algorithms we use are also *non-explicit*. That is, we will be showing that there is a low-space MPC algorithm for a specific task, but we will not be able to provide a procedure to explicitly design it (there is generally a brute-force procedure obvious from the proof, but one that requires at least exponential computation, and possibly also too much space to perform in low-space MPC). In this paper, non-uniform and non-explicit MPC algorithms will be occasionally used in the context of derandomization.

#### Component-stable MPC algorithms

Now, after our discussion in Sects. 2.1 to 2.3, we are ready to provide a new definition of component-stable MPC algorithms used in this paper.

##### Definition 5

(*Component-stable* *MPC* *algorithms*) A randomized MPC algorithm $A_{MPC}$ is **component-stable** if its output at any node *v* is entirely, deterministically, dependent on the *topology and IDs (but independent of names)* of *v*’s connected component (which we will denote *CC*(*v*)), *v* itself, the exact number of nodes *n* and maximum degree $\Delta$ in the *entire* input graph, and the input random seed ${\mathcal {S}}$. That is, the output of $A_{MPC}$ at *v* can be expressed as a deterministic function $A_{MPC}(CC(v),v,n,\Delta ,{\mathcal {S}})$.

A deterministic MPC algorithm $A_{MPC}$ is component-stable under the same definition, but omitting dependency on the random seed ${\mathcal {S}}$.

Finally, let us state the main technical result demonstrating the power of our revised framework of component-stable MPC algorithms, lifting unconditional lower bounds from the LOCAL model to conditional lower bounds for low-space component-stable MPC algorithms. The following theorem extends the main result in the component-stable algorithms framework due to Ghaffari et al. [26, Theorem I.4] to our framework and enhances it to include lower bounds against *deterministic algorithms*, and lower bounds with *dependency on maximum input degree* $\Delta$. Informally, similarly to [26, Theorem I.4], Theorem 5 below states that, conditioned on the connectivity conjecture, for *O*(1)-replicable graph problems, any $T(N,\Delta )$-round lower bound in the LOCAL model yields a $\Omega (\log T(N,\Delta ))$-round lower bound for any low-space component-stable MPC algorithm ${\mathcal {A}}_{\textsf {MPC}}$. Furthermore, the claim holds for both randomized and deterministic algorithms (deterministic algorithms were not studied in [26]).

The proof of Theorem 5 is given in Sect. 3 (the notion of constrained functions is defined in Definition 12).

##### Theorem 5

(Lifting LOCAL lower bounds to component-stable MPC algorithms) Let ${\mathcal {P}}$ be a *O*(1)-replicable graph problem that has a $T(N,\Delta )$-round lower bound in the randomized LOCAL model with shared randomness, for constrained function *T*, on graphs with input estimate *N* and maximum degree $\Delta$, from some normal family ${\mathcal {G}}$. Suppose that there is a randomized $o(\log T(n,\Delta ))$-round low-space component-stable MPC algorithm ${\mathcal {A}}_{\textsf {MPC}}$ for solving ${\mathcal {P}}$ on legal *n*-node graphs with maximum degree $\Delta$ from ${\mathcal {G}}$, succeeding with probability at least $1-\frac{1}{n}$. Then, there exists a low-space randomized MPC algorithm ${\mathcal {A}}^*$ that can distinguish one *n*-node cycle from two $\frac{n}{2}$-node cycles in $o(\log n)$ rounds, succeeding with probability at least $1-\frac{1}{n}$.

The same holds if the LOCAL lower bound and algorithm ${\mathcal {A}}_{\textsf {MPC}}$ are both deterministic (but the obtained algorithm ${\mathcal {A}}^*$ remains randomized).

### Landscape of MPC complexity classes and component-stability

In this section we define MPC complexity classes considered in this paper. We study the MPC model introduced in Sect. 1 and described in details Sect. 2.4, focusing on low-space MPC s.

Let us begin with reminding the reader a fundamental obstacle to fully understand the computational complexity of problems in the low-space MPC setting: a seminal work of Roughgarden et al. [44] showed that obtaining any unconditional lower bound in the low-space MPC setting ultimately leads to breakthrough results in circuit complexity. In view of that, we will take a more modest approach and will rely on *conditional* hardness results based on the widely believed *connectivity conjecture*. The core hardness result considered in this paper is a revised framework lifting unconditional lower bounds from the LOCAL model to conditional lower bounds for low-space component-stable MPC algorithms (see Sects. 2, 3, which amend the framework developed earlier in [26]). In particular, this framework can be used to obtain a number of deterministic and randomized lower bounds for low-space component-stable MPC algorithms (these bounds are conditioned on the connectivity conjecture). Therefore, providing low-space component-unstable MPC algorithms that beat these bounds will demonstrate the *conditional complexity gap* between low-space component-stable and component-unstable MPC algorithms—which is the ultimate goal of this section.

We focus on upper bound round complexity, local space, global space, and success probability which depend only on the number *n* of the graph’s nodes in the input instance from ${\mathbb {G}}$. Since our main focus is to study the low-space MPC regime, we consider the following definitions for MPC algorithms and MPC component-stable algorithms (see Sect. 2.4).

#### Definition 6

(*DetMPC*) We will denote by $\textsf {DetMPC} (T(n))$ the class of all graph problems for which there is a (possibly non-uniform and non-explicit) deterministic low-space MPC algorithm ${\mathcal {A}}$, such that for some positive constant *c*, algorithm ${\mathcal {A}}$ has round complexity at most $c \cdot T(n)$.

#### Definition 7

(*S-DetMPC*) We will denote by $\textsf {S-DetMPC} (T(n))$ the subclass obtained by restricting $\textsf {DetMPC} (T(n))$ to component-stable algorithms. That is, $\textsf {S-DetMPC} (T(n))$ is the class of all graph problems for which there is a (possibly non-uniform and non-explicit) deterministic low-space component-stable MPC algorithm ${\mathcal {A}}$, such that for some positive constant *c*, algorithm ${\mathcal {A}}$ has round complexity at most $c \cdot T(n)$.

#### Definition 8

(*RandMPC*) We will denote by $\textsf {RandMPC} (T(n))$ the class of all graph problems ${\mathcal {P}}$ for which there is a randomized low-space MPC algorithm ${\mathcal {A}}$, such that for some positive constant *c*, algorithm ${\mathcal {A}}$ solves ${\mathcal {P}}$ with probability $1 - \frac{1}{n}$ and has round complexity at most $c \cdot T(n)$.

#### Definition 9

(*S-RandMPC*) $\textsf {S-RandMPC} (T(n))$ is the subclass obtained by restricting $\textsf {RandMPC} (T(n))$ to component-stable algorithms. That is, $\textsf {S-RandMPC} (T(n))$ is the class of all graph problems ${\mathcal {P}}$ for which there is a randomized low-space component-stable MPC algorithm ${\mathcal {A}}$, such that for some positive constant *c*, algorithm ${\mathcal {A}}$ solves ${\mathcal {P}}$ with probability $1 - \frac{1}{n}$ and has round complexity at most $c \cdot T(n)$.

Clearly, observe that for any function *T*(*n*), we have both $\textsf {S-DetMPC} (T(n)) \subseteq \textsf {DetMPC} (T(n))$ and $\textsf {S-RandMPC} (T(n)) \subseteq \textsf {RandMPC} (T(n))$. However, as mentioned in Introduction, it has been informally argued (for example, by Ghaffari et al. [26]) that most, if not all, MPC algorithms are or can be easily made component-stable. Thus one could imagine that the pairs of sets $\textsf {S-DetMPC} (T(n))$ and $\textsf {DetMPC} (T(n))$, and $\textsf {S-RandMPC} (T(n))$ and $\textsf {RandMPC} (T(n))$ are in fact identical, especially in the regime of small *T*(*n*). However, we will demonstrate that (assuming the connectivity conjecture) this is not the case, and that some low-space general MPC algorithms can do better than their component-stable counterparts.

We begin with the study of *deterministic* complexity. Our first lemma shows that, informally, $\textsf {S-DetMPC} \ne \textsf {DetMPC}$ (when omitting functions *T*(*n*) during complexity class comparisons, we mean that there *exists* some *T*(*n*) for which the comparison holds, when both classes are parameterized by *T*(*n*)). In Sect. 4.2.2 we consider a sinkless orientation problem for which, on one hand, (assuming the connectivity conjecture) there is no deterministic low-space component-stable MPC algorithm running in $o(\log \log _\Delta n)$ rounds (Theorem 18) and, on the other hand, which can be solved by a low-space deterministic MPC algorithm running in $poly(\Delta )+O(\log \log \log n)$ rounds (Theorem 19), surpassing the component-stable lower bound for $\Delta = \log ^{o(1)}\log n$. In fact, these results hold even for forests. Similar results are also shown for some variants of edge-coloring (Theorems 20, 21) and vertex-coloring (Theorems 22, 23). Further, while the main deterministic upper bounds here use heavy local computation, for bounded degree graphs their local computation is ${\text {poly}}(n)$, demonstrating that *component-instability helps for deterministic algorithms even using polynomial computation*. (In Sect. 4.3, we demonstrate a similar result for the class of LOCAL *extendable* algorithms, showing that instability also helps in deterministic algorithms for two cornerstone problems in low-space MPC: maximal independent set and maximal matching.) This gives the following.

#### Theorem 6

There is some function *T*(*n*) such that, conditioned on the connectivity conjecture,

$$\begin{aligned} \textsf {S-DetMPC} (T(n)) \subsetneqq \textsf {DetMPC} (T(n)) \hspace{5.0pt}. \end{aligned}$$

Next, we move to the study of *randomized* algorithms and show that, informally, $\textsf {S-RandMPC} \ne \textsf {RandMPC}$. This result follows directly from our Theorem 1, which shows that some variant of the independent set problem has a deterministic low-space non-stable constant-round MPC algorithm and conditioned on the connectivity conjecture, there is no $o(\log \log ^* n)$-round low-space component-stable MPC algorithm that succeeds with probability at least $1 - \frac{1}{n}$.

#### Theorem 7

There is some function *T*(*n*) such that, conditioned on the connectivity conjecture,

$$\begin{aligned} \textsf {S-RandMPC} (T(n)) \subsetneqq \textsf {RandMPC} (T(n)) \hspace{5.0pt}. \end{aligned}$$

Next, we provide a conditional separation between randomized and deterministic algorithms (Theorem 13). This separation follows by combining (i) the conditional lifting for randomized component-stable algorithms and deterministic component-stable algorithm using the framework in Theorem 5 with (ii) local problems for which there is provable gap in their randomized and deterministic LOCAL complexity (e.g., [5, 11, 12]). This yields the following.

#### Theorem 8

There is some function *T*(*n*) such that, conditioned on the connectivity conjecture,

$$\begin{aligned} \textsf {S-DetMPC} (T(n)) \subsetneqq \textsf {S-RandMPC} (T(n)) \hspace{5.0pt}. \end{aligned}$$

Further, in Sect. 6, we study the power of deterministic low-space component-unstable MPC algorithms and their relationship to the randomized ones. In Lemma 32 we prove that if there is a randomized MPC algorithm that solves a graph problem ${\mathcal {P}}$ on *n*-node graphs with maximum degree $\Delta$ in $T(n,\Delta )$ rounds, then one can derandomized such algorithm to solve the same problem in $O(T(n,\Delta ))$ rounds. The resulting deterministic MPC algorithm is component-unstable, non-uniform, and non-explicit, and it has the same local space as that of the randomized MPC algorithm, and uses an $O(n^2)$-factor more machines. This yields the following.

#### Theorem 9

$$\textsf {DetMPC} (T(n)) = \textsf {RandMPC} (T(n))$$.

Let us summarize the results in this section, assuming the connectivity conjecture, and allowing non-uniform MPC algorithms. Our study demonstrates that for low-space MPC s, component-unstable algorithms are provably stronger than their component-stable counterparts, both for deterministic and randomized algorithms (Theorems 6, 7). Further, we see that for component-stable algorithms, randomized algorithms are provably stronger than their deterministic counterparts (Theorem 8), however, for arbitrary (possibly component-unstable) algorithms this is not the case: any randomized algorithm can be efficiently simulated by a deterministic one (Lemma 32 and Theorem 9).

## Conditional MPC lower bounds from LOCAL

In this section, we present a framework lifting unconditional lower bounds from the LOCAL model to conditional lower bounds for low-space component-stable MPC algorithms, extending and revising the analysis of Ghaffari et al. [26] to prove Theorem 5.

While on a high level, our analysis follows closely the approach from [26], our arguments diverge in several *subtle* but *crucial* places. On a technical level, we rely on the central but also quite subtle notions of *replicable graph problems*, *normal graph families*, $(D, \varepsilon , n, \Delta )$-*sensitive* MPC * algorithms*, and a revised definition of *component stability* (see Definition 5). The two major reasons behind these changes are:

- to make the arguments robust against the issues we have identified concerning component-stability, and incorporate the definitional changes that these issues necessitated, and
- to extend the arguments to include lower bounds against *deterministic algorithms*, and lower bounds with *dependency on maximum input degree* $\Delta$.

After introducing some useful notation in Sect. 3.1, we show in Sect. 3.2 that in our setting, for *R*-replicable graph problems for normal graph families, lower bounds for LOCAL algorithms imply the existence of some graphs which can be distinguished by component-stable MPC algorithms only by relying on non-local information. Then, in Sect. 3.3, we apply this non-locality to provide a conditional MPC lower bound for component-stable MPC algorithms for *O*(1)-replicable graph problems in our setting (for normal graph families). In particular, conditioned on the connectivity conjecture, our main result (Theorem 5) states, informally, that for *O*(1)-replicable graph problems, any $T(N,\Delta )$-round lower bound in the LOCAL model yields a $\Omega (\log T(N,\Delta ))$-round lower bound for any low-space component-stable MPC algorithm ${\mathcal {A}}_{\textsf {MPC}}$. Furthermore, the claim holds for both randomized and deterministic algorithms.

### Basic definitions: *normal graph families* and *sensitive*MPC* algorithms*

#### Definition 10

Two connected graphs $G = (V, E)$ and $G' = (V', E')$, with *center* nodes $v \in V$ and $v'\in V'$ respectively, are *D***-radius-identical** if the topologies and node IDs (but not necessarily names) of the *D*-radius balls around *v* and $v'$ are identical.

Our next definition (related to [26, Definition III.1], though set up in our framework), formalizes the notion of MPC algorithms depending on non-local information in the graph.

#### Definition 11

($(D, \varepsilon , n, \Delta )$-*sensitive* MPC *algorithms*) For integers $D,n,\Delta \ge 0$ and some $\varepsilon \in [0,1]$, a component-stable MPC algorithm ${\mathcal {A}}$ for some graph problem is called $(D, \varepsilon , n,\Delta )$**-sensitive** with respect to two *D*-radius-identical centered graphs $G = (V, E)$ and $G' = (V', E')$, with centers $v \in V$ and $v'\in V'$, if the probability (over choice of seed ${\mathcal {S}}$) that ${\mathcal {A}}(G, v, n, \Delta , {\mathcal {S}}) \ne {\mathcal {A}}(G', v', n, \Delta , {\mathcal {S}})$ is at least $\varepsilon$.

We can apply this definition also to deterministic component-stable algorithms. Since these do not depend on the random seed, any $(D, \varepsilon , n, \Delta )$-sensitive deterministic component-stable MPC algorithm with $\varepsilon > 0$ is $(D, 1, n, \Delta )$-sensitive.

As one final point of notation, in this section our arguments will often involve several different graphs. For clarity, for any graph *G* we will denote by $n_G$ its number of nodes.

### LOCAL hardness yields indistinguishability of graphs locally

Our next lemma (cf. [26, Lemma III.1]) shows that for any *R*-replicable graph problem, a lower bound for a normal graph family for any LOCAL algorithm implies some useful property for any component-stable MPC algorithm for that problem: to distinguish some graphs from that normal family one needs to rely on non-local information, in the sense of Definition 11.

#### Lemma 10

For any $N, \Delta , R \in {\mathbb {N}}$, let ${\mathcal {P}}$ be an *R*-replicable graph problem for which there is no $T(N,\Delta )$-round LOCAL algorithm (with shared randomness) that solves ${\mathcal {P}}$ on graphs from some normal family ${\mathcal {G}}$ of maximum degree $\Delta$, with input size estimate *N* (i.e., satisfying $n_H \le N \le poly(n_H)$), and with probability at least $1-\frac{1}{N}$.

Suppose there is a component-stable MPC algorithm ${\mathcal {A}}_{\textsf {MPC}}$ that solves ${\mathcal {P}}$ on all graphs $G \in {\mathcal {G}}$, with probability at least $1 - \frac{1}{n_G}$. Then, there are two $T(N,\Delta )$-radius-identical centered graphs $G, G' \in {\mathcal {G}}$ with at most *N* nodes and maximum degree (exactly) $\Delta$, such that ${\mathcal {A}}_{\textsf {MPC}}$ is $(T(N,\Delta ), \frac{1}{4N^2},N^{R+2},\Delta )$-sensitive with respect to $G,G'$.

The same claim holds if the LOCAL lower bound and algorithm ${\mathcal {A}}_{\textsf {MPC}}$ are both *deterministic*.

#### Proof

The proof is by contradiction. Denote $D = T(N,\Delta )$ and $\varepsilon = \frac{1}{4N^2}$. Let us assume that there are no two *D*-radius-identical centered graphs $G, G' \in {\mathcal {G}}$ with at most *N* nodes and maximum degree $\Delta$ such that the given MPC algorithm ${\mathcal {A}}_{\textsf {MPC}}$ is $(D, \varepsilon , N^{R+2}, \Delta )$-sensitive with respect to *G* and $G'$. We use this fact to construct a *D*-round randomized LOCAL algorithm ${\mathcal {A}}_{\textsf {LOCAL}}$ to solve ${\mathcal {P}}$ on graphs with valid size estimate *N* with probability at least $1 - \frac{1}{N}$, which is a contradiction to the assumption of the lemma.

***Design of the*** LOCAL  ***algorithm.***

The input for ${\mathcal {A}}_{\textsf {LOCAL}}$ is an unbounded random seed ${\mathcal {S}}$, a graph $H \in {\mathcal {G}}$, integers *N*, $\Delta$, and the assignment of the IDs to the nodes of *H*, such that:

- *N* is the input size estimate, that is, $n_H \le N \le poly(n_H)$,
- $\Delta$ is the maximum degree of *H*,
- all IDs are in $[{\text {poly}}(N)]$ and are component-unique.

For a fixed *H*, let us take an arbitrary node *v* and describe how to determine *v*’s output under ${\mathcal {A}}_{\textsf {LOCAL}}$, on a particular input ${\mathcal {I}} = \langle H, {\mathcal {S}} \rangle$. Node *v* first collects its *D*-radius ball $B_D(v)$ in *H*. It then considers the family ${\mathcal {H}}_v$ of all graphs with at most *N* nodes, centered at *v*, with component-unique IDs from $[{\text {poly}}(N)]$, and maximum degree $\Delta$ that are *D*-radius-identical to $B_D(v)$, i.e., the set of possible inputs to ${\mathcal {A}}_{\textsf {LOCAL}}$ for which *v* would see $B_D(v)$ as its *D*-radius ball. For each $G\in {\mathcal {H}}_v$, *v* creates a *simulation graph* $\Gamma _G$ (see Definition 4) as follows: $\Gamma _G$ consists of $\lfloor \frac{N^{R+2}}{n_G} \rfloor \ge n_G^R$ disjoint copies of *G*. One of these copies is arbitrarily designated the *“true” copy*, and its nodes use the IDs of *G* as their own IDs and names. All other copies use the same IDs, but as names they use arbitrary unique values in $[{\text {poly}}(N)]$. We also add enough isolated nodes to raise the number of nodes to exactly $N^{R+2}$ (i.e., $N^{R+2} - n_G\lfloor \frac{N^{R+2}}{n_G} \rfloor < n_G$ isolated nodes), sharing the same arbitrary ID, and with arbitrary unique names, in $[{\text {poly}}(N)]$. Note that this construction corresponds closely to Definition 4 of problem replicability, and is the reason we chose such a definition.

In order to determine the output of ${\mathcal {A}}_{\textsf {LOCAL}}$ at *v*, we will be running ${\mathcal {A}}_{\textsf {MPC}}$ on the simulation graph $\Gamma _G$. For the supply of randomness, denote ${\mathcal {S}}'$ to be ${\mathcal {S}}$ curtailed to the length required by ${\mathcal {A}}_{\textsf {MPC}}$, i.e., the first ${\text {poly}}(N)$ bits. Since ${\mathcal {A}}_{\textsf {MPC}}$ is component-stable, its output at *v* on $\Gamma _G$, using seed ${\mathcal {S}}'$, is ${\mathcal {A}}_{\textsf {MPC}}(G,v,N^{R+2},\Delta ,{\mathcal {S}}')$.

Then, we set ${\mathcal {A}}_{\textsf {LOCAL}} (H,v,{\mathcal {S}})$ to be

$$\begin{aligned} \arg \max _{x} \vert \{G\in {\mathcal {H}}_v: {\mathcal {A}}_{\textsf {MPC}}(G,v,N^{R+2},\Delta ,{\mathcal {S}}')=x \} \vert \hspace{5.0pt}, \end{aligned}$$

i.e., *v*’s output label output by ${\mathcal {A}}_{\textsf {LOCAL}}$ is that returned by the most ${\mathcal {A}}_{\textsf {MPC}}$ simulations.

***Correctness of the*** LOCAL ***algorithm***

We use our assumption that there are no two *D*-radius-identical centered graphs $G, G' \in {\mathcal {G}}$ with at most *N* nodes and maximum degree $\Delta$ for which ${\mathcal {A}}_{\textsf {MPC}}$ is $(D, \varepsilon , N^{R+2}, \Delta )$-sensitive with respect to *G* and $G'$. Hence, for any $G, G' \in {\mathcal {H}}_v$, with probability at least $1-\varepsilon$ over choice of ${\mathcal {S}}$, ${\mathcal {A}}_{\textsf {MPC}}(G, v, N^{R+2}, \Delta , {\mathcal {S}}')= {\mathcal {A}}_{\textsf {MPC}}(G', v, N^{R+2}, \Delta , {\mathcal {S}}')$. In particular, since the actual input graph *H* is certainly in ${\mathcal {H}}_v$,

$$\begin{aligned} {\textbf{E}}_{{\mathcal {S}}}\left[ \frac{ \vert \left\{ G\in {\mathcal {H}}_v: \begin{array}{c} {\mathcal {A}}_{\textsf {MPC}}(G,v,N^{R+2},\Delta ,{\mathcal {S}}')\\ \ne {\mathcal {A}}_{\textsf {MPC}}(H,v,N^{R+2},\Delta ,{\mathcal {S}}') \end{array} \right\} \vert }{ \vert {\mathcal {H}}_v \vert }\right]&\le \varepsilon \hspace{5.0pt}. \end{aligned}$$

Then, for the random seed ${\mathcal {S}}$, the probability that

$$\begin{aligned} \frac{ \vert \left\{ G\in {\mathcal {H}}_v:\begin{array}{c} {\mathcal {A}}_{\textsf {MPC}}(G,v,N^{R+2},\Delta ,{\mathcal {S}}') \\ \ne {\mathcal {A}}_{\textsf {MPC}}(H,v,N^{R+2},\Delta ,{\mathcal {S}}') \end{array} \right\} \vert }{ \vert {\mathcal {H}}_v \vert }&\ge \frac{1}{2} \hspace{5.0pt}. \end{aligned}$$

is at most $2\varepsilon$ by Markov’s inequality. We therefore have

$$\begin{aligned}&\textbf{Pr}\left[ {\mathcal {A}}_{\textsf {LOCAL}} (H, v, {\mathcal {S}}) = {\mathcal {A}}_{\textsf {MPC}}(H, v, N^{R+2}, \Delta , {\mathcal {S}}')\right] \\&\quad \ge 1 - 2 \varepsilon \hspace{5.0pt}. \end{aligned}$$

Taking a union bound over all nodes $v \in V(H)$, we have that

$$\begin{aligned}&\textbf{Pr}\left[ \text {for all }v \in V(H): \begin{array}{c} {\mathcal {A}}_{\textsf {LOCAL}} (H,v,{\mathcal {S}}) =\\ {\mathcal {A}}_{\textsf {MPC}}(H,v,N^{R+2},\Delta ,{\mathcal {S}}') \end{array}\right] \\&\quad \ge 1 - 2 N \varepsilon \hspace{5.0pt}. \end{aligned}$$

In this case, the overall output of ${\mathcal {A}}_{\textsf {LOCAL}} (H, {\mathcal {S}})$ is equal to the output of ${\mathcal {A}}_{\textsf {MPC}}$ on the true copy of *H* when run on $\Gamma _H$ with seed $S'$. Since $\Gamma _H \in {\mathcal {G}}$ (as it is a disjoint union of members of ${\mathcal {G}}$, see Definition 2), ${\mathcal {A}}_{\textsf {MPC}}$ returns a valid output on $\Gamma _H$ with probability $1-1/n_{\Gamma _H} = 1- N^{-(R+2)}$. Note that $\Gamma _H$ is a disjoint union of at least $n_H^{R}$ copies of *H* and fewer than $n_H$ extra isolated nodes. This satisfies the construction in the definition of *R*-replicability.^10^ Since ${\mathcal {A}}_{\textsf {MPC}}$ is component-stable, it must return the same output labeling in all copies of *H* in $\Gamma _H$ (since they share the same topology and IDs), and likewise must return the same output label on all isolated nodes. So, the output labeling given on $\Gamma _H$ is of the form required in the replicability definition, and so ${\mathcal {A}}_{\textsf {MPC}}$ returns a valid output on the true copy (and, indeed, on all copies) of *H* in $\Gamma _H$.

Then, by a union bound, ${\mathcal {A}}_{\textsf {LOCAL}}$’s output is correct with probability at least $1-2N\varepsilon - N^{-(R+2)} \ge 1 - \frac{1}{N}$, reaching a contradiction to our assumption of a LOCAL lower bound. This proves the randomized part of the lemma.

To prove the deterministic analogue, we perform exactly the same construction, except that ${\mathcal {A}}_{\textsf {LOCAL}}$ does not have the random seed ${\mathcal {S}}$, and does not pass it to ${\mathcal {A}}_{\textsf {MPC}}$. Since our algorithm ${\mathcal {A}}_{\textsf {LOCAL}}$ is now deterministic, we have ${\mathcal {A}}_{\textsf {LOCAL}}(H,v) = {\mathcal {A}}_{\textsf {MPC}}(H,v,N^{R+2},\Delta )$ with certainty for all *v*, i.e., ${\mathcal {A}}_{\textsf {LOCAL}}$’s output is identical to ${\mathcal {A}}_{\textsf {MPC}}$’s output on an $N^{R+2}$-node graph containing *H* as a connected component. Since ${\mathcal {A}}_{\textsf {MPC}}$ is also deterministic, this is a valid output on *H* with certainty. We have then constructed a deterministic LOCAL algorithm solving ${\mathcal {P}}$, contradicting our initial assumption. So, we must instead have that ${\mathcal {A}}_{\textsf {MPC}}$ is $(T(N,\Delta ), 1,N^{R+2},\Delta )$-sensitive, and therefore, since it is deterministic, $(T(N,\Delta ), 1,N^{R+2},\Delta )$-sensitive. $\square$

### Conditional MPC lower bounds from LOCAL (proving Theorem 5)

In this section we apply Lemma 10 to obtain conditional lower bounds for component-stable MPC algorithms for *O*(1)-replicable graph problems through their hardness in the LOCAL model. Our main result, Theorem 5, can be seen as a revision and extension of the main arguments due to Ghaffari et al. [26] in their Theorem I.4, applied to low-space component-stable MPC algorithms for *O*(1)-replicable graph problems and for normal graph families.

Since the complexity of our algorithms depends on two parameters, input size estimate *N* and maximum degree $\Delta$, we define the type of functions for which we will study lower bounds:

#### Definition 12

We call a function $T: {\mathbb {N}}\times {\mathbb {N}}\rightarrow {\mathbb {N}}$ **constrained** if $T(N,\Delta ) = O(\log ^\gamma N)$ for all $\Delta \le N$ and $\gamma \in (0,1)$, and $T(N^c,\Delta ) \le c \cdot T(N,\Delta )$ for any $c\ge 1$.

This is similar to the corresponding requirement $T(N) = O(\log ^\gamma N)$ in [26], but allowing for dependency on $\Delta$. The definition of constrained functions further incorporates a ‘smoothness’ condition, which is also implicitly required by [26] (there, it is used that when $T(N) = O(\log ^\gamma N)$, $T({\text {poly}}(N)) = O(T(N))$; while this is true for smooth functions, it is not technically true in general).^11^

With the notion of constrained functions at hand, we can leverage Lemma 10 to relate the complexity of LOCAL algorithms for *R*-replicable graph problems in normal graph families to the connectivity conjecture. In particular, given the conjectured hardness of this problem in the MPC model, this will imply (see Theorem 5 for a precise statement) a lower bound of $\Omega (\log T(n,\Delta ))$ rounds for any low-space component-stable MPC algorithm for any *O*(1)-replicable graph problem in normal graph families with no $T(N,\Delta )$-round LOCAL algorithm.

We begin with an auxiliary lemma that relates Lemma 10 to the complexity of low-space MPC algorithms for the *D*-diameter *s*-*t* connectivity problem. The *D-diameter* *s*-*t* *connectivity problem* is formally defined in [26, Definition IV.1] as follows: Given two special nodes *s* and *t* in the graph, the goal is to determine whether *s* and *t* are connected or not, in a way that provides the following guarantee: If *s* and *t* are in the same connected component and that component is a path of length at most *D* with *s* and *t* at its two endpoints, then the algorithm should output **YES**, with probability $1 - \frac{1}{{\text {poly}}(n)}$. If *s* and *t* are in different connected components, the algorithm should output **NO**, with probability $1 - \frac{1}{{\text {poly}}(n)}$. In other cases, the output can be arbitrary.

#### Lemma 11

Let ${\mathcal {P}}$ be a *O*(1)-replicable graph problem that has a $T(N,\Delta )$-round lower bound in the randomized LOCAL model with shared randomness, for constrained function *T*, on graphs with input size estimate *N* and maximum degree $\Delta$, from some normal family ${\mathcal {G}}$. Suppose that there is a randomized $o(\log T(n,\Delta ))$-round low-space component-stable MPC algorithm ${\mathcal {A}}_{\textsf {MPC}}$ for solving ${\mathcal {P}}$ on legal *n*-node graphs with maximum degree $\Delta$ from ${\mathcal {G}}$, succeeding with probability at least $1-\frac{1}{n}$. Then, there exists a low-space randomized MPC algorithm $B_{\text {st-conn}}$ with round complexity $o(\log T(n,\Delta ))$ for $T(n,\Delta )$-diameter *s*-*t* connectivity on graphs with *n* nodes, succeeding with probability at least $1-\frac{1}{n}$.

The same holds if the LOCAL lower bound and algorithm ${\mathcal {A}}_{\textsf {MPC}}$ are both deterministic (but the obtained MPC algorithm $B_{\text {st-conn}}$ is still randomized).

One point of note in the theorem statement is that we do not make any constraint on the maximum degree of the *s*-*t* connectivity instance; indeed, for that problem any node of degree over 2 can be immediately discarded, so maximum degree is largely irrelevant. $\Delta$ appears only in the expression for the diameter *D*.

#### Proof

Let *c* be a sufficiently large constant. We will require that ${\mathcal {P}}$ is $\frac{c}{\phi }$-replicable; since replicability is monotone, this will be the case when *c* is sufficiently large. We then construct our randomized MPC algorithm for $T(n,\Delta )$-diameter *s*-*t* connectivity $B_{\text {st-conn}}$ as follows:

We are given an *n*-node input graph *H*, and ${\text {poly}}(n)$-bit random seed ${\mathcal {S}}$, for $T(n,\Delta )$-diameter *s*-*t* connectivity. We set $N=n^{\phi /2}$, denote $D:= T(N,\Delta )$, and apply Lemma 10 to obtain^12^ two *D*-radius-identical centered graphs $G,G'\in {\mathcal {G}}$ with at most *N* nodes and maximum degree $\Delta$, such that ${\mathcal {A}}_{\textsf {MPC}}$ is $(D, \frac{1}{4N^2},n^{c/\phi +2},\Delta )$-sensitive with respect to $G,G'$.

Our algorithm $B_{\text {st-conn}}$ will work by combining the results of several parallel simulations, run on disjoint groups of machines. We first divide the seed ${\mathcal {S}}$ equally among these simulations; by choosing our input ${\text {poly}}(n)$ seed length sufficiently high, we can guarantee that each simulation is given sufficient randomness. In each simulation, each node in $v\in V(H)$ uses part of the random seed assigned to the simulation to independently, uniformly randomly, choose a value $h(v) \in [D]$.

For each simulation we now construct a pair of graphs $G_H$ and $G'_H$ on which to run ${\mathcal {A}}_{\textsf {MPC}}$. In each case we begin with the graph *H*, and discard all nodes of degree higher than 2. Then, for each remaining node in *H* we collect its radius-1 neighborhood onto a machine (this takes *O*(1) rounds and does not increase asymptotic space usage). Each node of *H* then drops out if its radius-1 neighborhood is not consistent with being part of an $s-t$ path with consecutively increasing values of *h* along the path (other than the value of *h*(*t*), which we make no requirements for). In particular:

- Nodes *s* and *t* in *H* must have degree 1 (and otherwise we terminate the simulation immediately and output **NO**).
- Other nodes must have degree 2, and the values of *h* in their radius-1 neighborhood must be a consecutive triplet (other than *h*(*t*)).

Now, every remaining node (i.e., satisfying these conditions) in *H* is assigned (copies of) nodes in *G* and $G'$ as follows:

- Node *s* in *H* is assigned all nodes of distance at most *h*(*s*) from *v* in *G* and $G'$.
- Node *t* in *H* is assigned all nodes of distance more than *D* from *v* in *G* and $G'$.
- Other nodes *u* in *H* are assigned the nodes of distance exactly *h*(*u*) from *v* in *G* and $G'$.

We can now define the new graphs $G_H$ and $G'_H$ as follows:

The node sets of $G_H$ and $G'_H$ consist of every assigned copy of nodes in *G* and $G'$ respectively. We also add to $G_H$ and $G'_H$ an extra, full, copy of *G*, disconnected from the rest of the graph, whose sole purpose is to ensure that the maximum degree of $G_H$ and $G'_H$ is exactly $\Delta$, and enough isolated nodes to raise the number of nodes (currently $O(nN) = O(n^{1+\phi /2})$) to exactly $n^{c/\phi +2}$ (which is larger for sufficiently large constant *c*). Let $w_{u}$ in $G_H$ denote a copy of *w* in *G*, assigned to node *u* in *H*. We place an edge between $w_{u}$ and ${\hat{w}}_{{\hat{u}}}$ in $G_H$ iff $w\sim _G {\hat{w}}$ and $u\sim _H {\hat{u}}$ (or $u={\hat{u}}$). As IDs, for a simulated node $w_{u}$, we use the original ID of *w*. We will show that these IDs remain component-unique in $G_H$ and $G'_H$. As names, we use (*name*(*u*), *name*(*w*)); these names are guaranteed to be fully unique. We arbitrarily choose fully unique names and IDs for the extra isolated nodes and copy of *G*.

The graphs $G_H$ and $G'_H$ can easily be constructed in *O*(1) rounds of MPC, using $O(n^{\phi })$ local space, since machines each need only store *G*, $G'$, and $O(n^{\phi })$ edges of *H*. We construct $G'_H$ from $G'$ analogously.

We see that the connected components of $G_H$ and $G'_H$ are isomorphic to induced subgraphs of *G* and $G'$ respectively. Furthermore, the IDs of nodes in these components are identical to the relevant subgraphs of *G* and $G'$, and are therefore component-unique (as they were in *G* and $G'$). In particular, we consider the topology and IDs of the connected components $CC({v_{s}})$ and $CC'(v_{s})$ containing $v_{s}$ in $G_H$ and $G'_H$ respectively:

1. If *s* and *t* are endpoints of a path of $p\le D+1$ *nodes* (and therefore of *length* at most *D*) in *H*, $h(s)=p-D$, and $h(u_d) = p-D+d$ for each $u_d\ne t$ which is the node at distance *d* along the path from *s*, then $CC({v_{s}}) = G$ and $CC'(v_{s}) = G'$;
2. otherwise, $$CC(v_{s})=CC'(v_{s})$$.

In the first case, this is because the nodes on the $s-t$ path are together assigned precisely one copy of each on the nodes in *G* and $G'$, in such away that every two adjacent nodes in *G* and $G'$ are assigned to adjacent nodes on this path. In the second case, the only nodes that differ in *G* and $G'$ are assigned only to *t*, and no outputs of *h* can cause these to be connected to $v_{s}$. Therefore the graphs of nodes that *are* connected to $v_{s}$ are identical.

We now simulate ${\mathcal {A}}_{\textsf {MPC}}$ on $G_H$ and $G'_H$ using a sufficiently large ${\text {poly}}(n)$-length part of ${\mathcal {S}}$ allocated to this simulation as the random seed (the same seed for both $G_H$ and $G'_H$). Since $G_H$ and $G'_H$ are in the *normal* family ${\mathcal {G}}$ (they are disjoint unions of induced subgraphs of $G, G'\in {\mathcal {G}}$), these are valid inputs for ${\mathcal {A}}_{\textsf {MPC}}$.

If *s* and *t* are endpoints of a path of length at most *D* in *H*, then the probability that all nodes *v* on this path choose the ‘correct’ value *h*(*v*) (i.e., as specified in point 1 above) is at least $D^{-D} = N^{-o(1)}$. In this case, we have $CC({v_{s}}) = G\in {\mathcal {G}}$ and $CC'(v_{s}) = G'\in {\mathcal {G}}$. Since ${\mathcal {A}}_{\textsf {MPC}}$ is component-stable, its output for $v_{s}$ in each case is equal to ${\mathcal {A}}_{\textsf {MPC}}(G,v,n^{c/\phi +2},\Delta ,{\mathcal {S}})$ and ${\mathcal {A}}_{\textsf {MPC}}(G',v,n^{c/\phi +2},\Delta ,{\mathcal {S}})$ respectively, and by sensitivity, with probability at least $\frac{1}{4N^2}$, these are different. If this occurs, the simulation output, and the overall output, is correctly **YES**.

If *s* and *t* are not endpoints of a path of length at most *D* in *H*, then in all simulations we have $CC({v_{s}})=CC'({v_{s}})$. Since ${\mathcal {A}}_{\textsf {MPC}}$ is component-stable, its output depends (deterministically) on these connected components (as well as equal values of $\Delta$, random seed ${\mathcal {S}}$, and $n_{G_H}=n_{G'_H}=n^{c/\phi +2}$), and is therefore the same in both cases. The output of all simulations is then correctly **NO**.

The final output for $B_{\text {st-conn}}$ is as follows: after running ${\text {poly}}(n)$-many concurrent simulations, we return **YES** if any simulation returned **YES**, and **NO** otherwise. We have already shown that if *s* and *t* are not endpoints of a path of length at most *D* in *H*, all simulations will return **NO**, so $B_{\text {st-conn}}$ will correctly output **NO**. It remains to show that $B_{\text {st-conn}}$ is also correct with high probability on **YES** instances.

Each simulation has a $D^{-D} = N^{-o(1)}$ probability of the nodes in the s-t path choosing the correct values of *h*(*v*), and a $\frac{1}{4N^2}$ probability of returning different outputs on $G_H$ and $G'_H$. Therefore, there is at least a $\frac{1}{4N^3}$ probability of the simulation returning **YES**. Since simulations use independent randomness, and we can run sufficiently large ${\text {poly}}(n)$ many of them in parallel, we can amplify the total probability of success to $1-\frac{1}{n}$ as required.

The round complexity of $B_{\text {st-conn}}$ is dominated by simulating ${\mathcal {A}}_{\textsf {MPC}}$ on graphs with $O(n^{c/\phi +2})$ nodes and maximum degree $\Delta$, which, using that *T* is a constrained function, is $O(T(n^{c/\phi +2},\Delta )) = O(T(n,\Delta ))$. $\square$

While Lemma 11 already provides a strong conditional lower bound for *O*(1)-replicable graph problems in our setting, the lower bound is conditioned on the complexity of solving *D*-diameter *s*-*t* connectivity. However, we can apply the framework [26] to extend the analysis to condition on the more standard connectivity conjecture—to conclude our analysis with the result stated in Theorem 5.

#### Proof of Theorem 5

This reduction from Lemma 11 is taken directly from Section V in [26]. First, we notice that [26, Lemma IV.1] applies to any MPC algorithm, i.e., is not affected by our changes to the definition of component-stability. Therefore, assuming that any low-space MPC algorithms distinguishing one *n*-node cycle from two $\frac{n}{2}$-node cycles requires $\Omega (\log n)$ rounds, we obtain that any low-space MPC algorithm that solves *D*-diameter *s*-*t* connectivity for $D \le \log ^\gamma n$ for a constant $\gamma \in (0, 1)$ requires $\Omega (\log D)$ rounds. By setting $D = T(n,\Delta )$, we can combine this fact with Lemma 11 to conclude the proof of the theorem. $\square$

### Applications of Theorem 5: conditional MPC lower bounds

Similarly as was been demonstrated by Ghaffari et al. [26], the lifting arguments in Theorem 5, when combined with the known lower bounds for LOCAL algorithms, lead to a host of conditional lower bounds for low-space component-stable MPC algorithms for many natural graph problems. In particular, we can recover the main applications of Ghaffari et al. [26] (see Theorems I.1, I.2, and I.3 there):

#### Theorem 12

Assuming the connectivity conjecture (that no low-space randomized MPC algorithm can distinguish one *n*-node cycle from two $\frac{n}{2}$-node cycles in $o(\log n)$ rounds, with failure probability at most $\frac{1}{n}$), the following lower bounds hold for randomized low-space component-stable MPC algorithms:

- $\Omega (\log \log n)$ rounds for a constant approximation of maximum matching (even on forests), a constant approximation of vertex cover, a maximal independent set;
- $\Omega (\log \log \log n)$ rounds for $(\Delta +1)$-coloring, unless the deterministic complexity of $(\Delta +1)$-coloring in the LOCAL model is $2^{\log ^{o(1)}\log n}$;
- $\Omega (\log \log \log n)$ rounds for the Lovász Local Lemma problem, or even for the special case of sinkless orientation where we should orient the edges of a *d*-regular graph, where $d \ge 4$, such that each node has at least one outgoing edge.

#### Proof

First, we notice that Ghaffari et al. [26] proved the following lower bounds for the randomized LOCAL model with shared randomness:

- $\Omega (\sqrt{\log n/\log \log n})$ rounds for a ${\text {polylog}}(n)$-approximate solution for the minimum vertex cover, maximum matching, or minimum dominating set problem, and for finding a maximal matching (even on trees) or a maximal independent set (Theorem V.1 and Corollary V.2 in [26]);
- $\Omega (\sqrt{\log \log n})$ rounds for $(\Delta +1)$-coloring unless the deterministic complexity of $(\Delta +1)$-coloring in the LOCAL model is $o(\log n)$ (indirectly in Corollary V.4 in [26]);
- $\Omega (\log \log n)$ rounds for the Lovász Local Lemma problem, or even for the special instance known as sinkless orientation where we should orient the edges of a *d*-regular graph, where $d \ge 4$, such that each node has at least one outgoing edge [26, Corollary V.5].

The proof of Theorem 12 now follows immediately by combining these LOCAL lower bound to Theorem 5, by taking each time ${\mathcal {G}}$ to be the set of all graphs, and by noticing that all the problems considered are *O*(1)-replicable (see Sect. 2.3.1, and for example, Lemma 2 for LCL s and Lemma 4 for approximate maximum matching). The only issue worth mentioning is that since the class of trees is not normal (see Definition 2), the lifting of the LOCAL lower bound for finding a maximal matching on trees extends only to forests, that is, that any randomized low-space component-stable MPC algorithm that returns a constant approximation of maximum matching on forests has $\Omega (\log \log n)$ rounds complexity. $\square$

Comparing the results claimed by Ghaffari et al. [26] (Theorems I.1, I.2, and I.3) with those proven in Theorem 12, there are only two differences: firstly, our lower bound for matching holds only for forests, while [26] claimed it for trees (for discussion of the reason for this difference, see Sect. 2.2), and secondly, our result for $(\Delta +1)$-coloring is updated to hold on a weaker conjecture of no $2^{\log ^{o(1)}\log n}$-round deterministic LOCAL algorithm for $(\Delta +1)$-coloring (since the original result in [26] conditioned on the $2^{o(\sqrt{\log n})}$-deterministic hardness, which was recently disproved in [45]).

#### Deterministic conditional MPC lower bounds

Finally, observe that Theorem 5 can be directly combined with existing deterministic LOCAL lower bounds to obtain deterministic lower bounds for low-space deterministic MPC algorithms. (Note that unlike in the case of randomized MPC algorithms above, one can directly apply deterministic LOCAL lower bounds as black-box in Theorem 5, since the issue of shared randomness is of no importance for deterministic algorithms.) For example, we apply it in Theorem 26 to give $\Omega (\log \Delta +\log \log n)$-round lower bounds for deterministic low-space component-stable MPC algorithms for maximal matching or maximal independent set (conditioned on the connectivity conjecture). Similarly, we present deterministic conditional lower bounds of $\Omega (\log \log _\Delta n)$ rounds for low-space component-stable MPC algorithms for sinkless orientation, $(2\Delta -2)$-edge coloring, and $\Delta$-vertex coloring, holding even in forests (Theorems 18, 20, 22). For some more lower bounds to which the framework is applicable, see, e.g., the recent deterministic LOCAL lower bounds for ruling sets in [6].

## Separation between stable and unstable deterministic MPC

We start by presenting a general statement characterizing local problems for which component-unstable MPC algorithms provably help. Specifically, this includes problems for which there is a provable exponential gap between their LOCAL deterministic and randomized complexities (e.g., as shown in [5, 11, 12]). The theorem is restricted to graphs of bounded (i.e., *O*(1)) degree, but this is still sufficient to demonstrate an exponential gap for many problems, since many LOCAL lower bounds are proven on bounded-degree graphs.

### Theorem 13

Let ${\mathcal {P}}$ be a *O*(1)-replicable graph problem. Let $T_r(N)$ and $T_d(N)$ be the randomized and deterministic, respectively, LOCAL round complexity of ${\mathcal {P}}$ on bounded-degree graphs with *n* nodes, with an input size estimate *N* and exact knowledge of $\Delta$. If $T_r(N) < \log ^{\gamma } N$ for some constant $\gamma \in (0,1)$, and if there is a provable exponential gap between $T_r(N)$ and $T_d(N)$, then, assuming the connectivity conjecture, there is an exponential gap between component-stable and component-unstable deterministic low-space MPC complexities for solving ${\mathcal {P}}$.

### Proof

Since $T_r(N) < \log ^{\gamma } N$ for some constant $\gamma \in (0,1)$ (see Definition 12), by Theorem 5, conditioning on the connectivity conjecture, any deterministic low-space *component-stable* MPC algorithm for ${\mathcal {P}}$ requires $\Omega (\log (T_d(n)))$ rounds. We will show that there exists a deterministic low-space *component-unstable* MPC algorithm for ${\mathcal {P}}$ running in $O(\log (T_r(n)))$ rounds using an $n^2$ factor more machines (the algorithm is possibly non-uniform and non-explicit).

We use Lemma 31, which says that it is sufficient to show a randomized low-space MPC algorithm for ${\mathcal {P}}$ that runs in $O(\log (T_r(n)))$ rounds and succeeds with probability at least $1-2^{-n^2}$. The algorithm employs $n^2$ parallel repetitions of procedure ${\mathcal {B}}$, each uses a disjoint set of machines and a distinct random coins determined by a shared random string of length ${\text {poly}}(n)$. The final output is determined by picking the *most successful* repetition (i.e., with the maximal number of legal node outputs). Procedure ${\mathcal {B}}$ simply collects the $2T_r(n)$ ball of each node $u \in V(G)$, which can be done in $O(\log (T_r(n)))$ rounds. The machines then simulate the randomized LOCAL algorithm on balls using the shared seed. This completes the description of the simulation.

We first show that this algorithm succeeds with probability $1-2^{-n^2}$. By the properties of the LOCAL algorithm, a single repetition succeeds with probability at least $1-\frac{1}{n}$ (over the randomness of the shared seed). Thus, by employing $n^2$ independent repetitions, we get the probability that all these repetitions fail is less than $2^{-n^2}$.

Notice that since $T_r(n) = o(\log n)$ and the input graph is bounded-degree, the $2T_r(n)$-ball of each node indeed fits the local space of each machine, and thus the MPC algorithm can be implemented with $n^{o(1)}$ local space, and hence is low-space. Finally, let us observe that the obtained algorithm is *component-unstable*, since it relies on globally agreeing on the outcome of all repetitions. $\square$

Notice that Theorem 13 implies Theorem 6, that is, demonstrates that, assuming the connectivity conjecture, there are some graph problems for which there are deterministic low-space component-unstable MPC algorithms that are provably faster than their component-stable counterparts. However, the weakness of Theorem 13 is that the obtained deterministic low-space component-unstable MPC algorithms are *non-uniform*. In the rest of this section we will address this issue and show that a similar claim holds also for uniform deterministic MPC algorithms, and those which use almost-optimal global space.

### Derandomization tools

In this section we present some basic derandomization tools used in our analysis.

#### Strongly $(\varepsilon ,k)$-wise independent hash functions

We will use the notion of *strongly* $(\varepsilon ,k)$-*wise independent hash functions* which are defined as follows:

##### Definition 13

Let *A* and *B* be two sets and let $\varepsilon \ge 0$. A family ${\mathcal {H}}$ of functions $A \rightarrow B$ is **strongly** $(\varepsilon ,k)$**-wise independent** if, when $h \in {\mathcal {H}}$ is chosen uniformly at random, for any $t \le k$ and for any *t* distinct $x_1, \dots , x_t \in A$ and any (not necessarily distinct) $y_1, \dots , y_t \in B$, we have

$$\begin{aligned} \left| \textbf{Pr}\left[ h(x_i) = y_i, 1 \le i \le t\right] - \frac{1}{ \vert B \vert ^t}\right|&\le \varepsilon \hspace{5.0pt}. \end{aligned}$$

That is, the probability of any particular $t\le k$ outputs of a random function from ${\mathcal {H}}$ differs from what one would expect under uniform full randomness only by $\varepsilon$. In our applications, we will choose $\varepsilon = n^{-c}$ for sufficiently large constant *c*, and we can then assume that these outputs are indeed fully independent (since the statistical difference is far below the failure probability of our algorithms).

Kurosawa et al. [35] presented the following result showing the existence of strongly $(\varepsilon ,k)$-wise independent families of size (and therefore requiring few bits to specify):

##### Theorem 14

[35] For any sets *A*, *B*, positive integer *k*, and positive real $\varepsilon$, there exists a strongly $(\varepsilon ,k)$-wise independent family ${\mathcal {H}}$ of size

$$\begin{aligned} 2^{O(\log \log \vert A \vert + k \log \vert B \vert + \log \frac{1}{\varepsilon })} \hspace{5.0pt}. \end{aligned}$$

Furthermore, each $h\in {\mathcal {H}}$ can be specified and evaluated on any input in ${\text {polylog}}( \vert A \vert , \vert B \vert ,2^k,\frac{1}{\varepsilon })$ time and space.

##### Proof

As noted in the proof of Theorem 2.9 of [35], by applying propositions 1.2 and 2.3 of [35] an $(\varepsilon ,k)$-wise independent family of the desired size can be constructed from the independent sample spaces of Alon et al. [1]. $\square$

#### Pseudorandom generators

A *Pseudorandom Generator (PRG)* is a function that gets a short random seed and expands it to a long one which look random, in the sense that it is indistinguishable from a random seed of the same length for such a formula.

##### Definition 14

(*Computational indistinguishability, Definition 7.1 in* [46]) Random variables *X* and *Y* taking values in $\{0,1\}^m$ are $(t,\varepsilon )$ **indistinguishable** if for every non-uniform algorithm *T* running in time at most *t*, we have $\vert \Pr [T(X)=1]-\Pr [T(Y)=1] \vert \le \varepsilon$.

##### Definition 15

(*PRG, Definition 7.3 in* [46]) A deterministic function ${\mathcal {G}}:\{0,1\}^d \rightarrow \{0,1\}^m$ is an $(t,\varepsilon )$ **pseudorandom generator (PRG)** if (1) $d < m$, and (2) ${\mathcal {G}}(U_d)$ and $U_m$ are $(t,\varepsilon )$ indistinguishable.

The following proposition uses the probabilistic method to show the existence of good PGRs.

##### Proposition 15

(Proposition 7.8 in [46]) For all $m \in {\mathbb {N}}$ and $\varepsilon >0$, there exists a (non-explicit) $(m,\varepsilon )$ pseudorandom generator ${\mathcal {G}}: \{0,1\}^d \rightarrow \{0,1\}^m$ with seed length $d = \Theta (\log m+\log (1/\varepsilon ))$.

We next notice that $(m,\varepsilon )$ PRGs of Proposition 15 can be computed by exhaustive search.

##### Lemma 16

(Time and space complexity of perfect PRGs) For all $m \in {\mathbb {N}}$ and $\varepsilon >0$, there exists an algorithm for computing the $(m,\varepsilon )$ PRG of Proposition 15 in time $\exp ({\text {poly}}(m/\varepsilon ))$ and space ${\text {poly}}(m/\varepsilon )$.

##### Proof

Our goal is to find an $(m,\varepsilon )$ PRG from $\{0,1\}^d$ to $\{0,1\}^m$ with seed length $d = \Theta (\log m+\log 1/\varepsilon )$, and the existence of one such PRG follows from Proposition 15.

The procedure for computing the PRG iterates over all different functions ${\mathcal {G}}: \{0,1\}^d \rightarrow \{0,1\}^m$ according to some fixed order. For each fixed function ${\mathcal {G}}: \{0,1\}^d \rightarrow \{0,1\}^m$, it iterates over all non-uniform algorithms ${\mathcal {A}}$ running in time *m* according to some fixed order, as well. To examine if ${\mathcal {G}}$ $\varepsilon$-fools ${\mathcal {A}}$ (in the sense of Definition 15), it is required to compare the output distribution of ${\mathcal {A}}$ run on a random *m*-bit string to the *m*-bit pseudo-random string obtained by evaluating the function ${\mathcal {G}}$ on a random *d*-bit vector. This is done by computing the output of algorithm ${\mathcal {A}}$ under each ${\mathcal {G}}(x)$ for every $x \in \{0,1\}^d$, as well as evaluating the algorithm ${\mathcal {A}}$ under each $y \in \{0,1\}^m$. The procedure picks the PRG function ${\mathcal {G}}^*:\{0,1\}^d \rightarrow \{0,1\}^m$ that $\varepsilon$-fools all *m*-time algorithms; the existence of one such ${\mathcal {G}}^*$ follows from Proposition 15.

We next bound the time and space complexity of this procedure. There are $(2^m)^{2^d} = 2^{O(m2^d)}$ different functions ${\mathcal {G}}$ mapping *d* bits into *m* bits, and there are $2^{O(m \log m)}$ non-uniform algorithms running in time *m* (i.e., Boolean circuits of size *m*). The time complexity is bounded by $2^{O(m2^d)} \cdot 2^{O(m \log m)} \cdot 2^d \cdot 2^d = 2^{{\text {poly}}(m/\varepsilon )}$.

We next bound the space complexity. To iterate over all the possible PRG candidates ${\mathcal {G}}: \{0,1\}^d \rightarrow \{0,1\}^m$, one needs to store the index of the current candidate function and its representation, which uses $O(m \cdot 2^d)$ bits. To iterate over all the *m*-time algorithms according to some fixed order, one needs to store the index of the current ${\mathcal {A}}$, and its representation which can be done with $O(m \log m)$ bits. Fixing the candidate PRG function to ${\mathcal {G}}$ and the given *m*-algorithm to ${\mathcal {A}}$, the evaluation of the algorithm ${\mathcal {A}}$ under each ${\mathcal {G}}(x)$ for every $x \in \{0,1\}^d$ can be done using $O(m \cdot 2^d)$ space. Finally, in order to evaluate ${\mathcal {A}}$ under each $y \in \{0,1\}^m$, it is sufficient to store the current index of the vector *y* considered. Altogether, the space requirements is $O(m \cdot 2^d)$; since $d = \Theta (\log m+\log (1/\varepsilon ))$, this is ${\text {poly}}(m/\varepsilon )$. $\square$

### Problems related to the Lovász local Lemma

We first demonstrate a deterministic complexity separation between component-stable and component-unstable algorithms for a group of problems related to the distributed Lovász Local Lemma: sinkless orientation, $(\Delta +o(\Delta ))$-edge coloring, and $o(\Delta )$-coloring triangle-free graphs.

These problems are known to be hard in the LOCAL model via proofs based on the *round elimination* technique [11]. We show that by derandomizing an algorithm for the constructive Lovász Local Lemma and plugging this result into known algorithms for the problems, we can surpass the component-stable lower bounds we obtain when lifting the LOCAL lower bounds to low-space MPC.

#### Algorithmic Lovász local lemma

We first present a deterministic low-space component-unstable MPC algorithm for the Lovász Local Lemma which uses *heavy local computations* to obtain a small number of MPC rounds (though for bounded-degree graphs, computation is still polynomial in *n*). Later we will demonstrate that this algorithm can be applied to obtain similar MPC algorithms for sinkless orientation, edge-coloring, and vertex-coloring.

The *algorithmic Lovász Local Lemma (LLL)* is defined as follows:

##### Definition 16

Consider a set ${\mathcal {V}}$ of independent random variables, and a family ${\mathcal {X}}$ of *n* (bad) events on these variables. Each event $A \in {\mathcal {X}}$ depends on some subset ${\mathcal {V}} (A) \subseteq {\mathcal {V}}$ of variables. Define the dependency graph $G_{\mathcal {X}} = ({\mathcal {X}} , \{(A, B) \mid {\mathcal {V}} (A) \cap {\mathcal {V}} (B) \ne \emptyset \})$ that connects any two events which share at least one variable. Let *d* be the maximum degree in this graph, i.e., each event $A \in {\mathcal {X}}$ shares variables with at most *d* other events $B \in {\mathcal {X}}$. Finally, define $p = \max _{A\in {\mathcal {X}} }\textbf{Pr}\left[ A\right]$.

The Lovász Local Lemma shows that $\textbf{Pr}\left[ \cap _{A\in {\mathcal {X}} } {\bar{A}}\right] > 0$ (i.e., there is some assignment of variables that does not satisfy any of the bad events) under the LLL criterion that $epd \le 1$. Our algorithmic goal is to *find* such an assignment of the variables (possibly under a stronger criterion).

We give the following deterministic low-space MPC algorithm.

##### Lemma 17

Any LLL instance with $d=\log ^{o(1)} n$ and $p\le \frac{1}{C} d^{-C}$ (for sufficiently high constant *C*), in which each bad event has ${\text {poly}}(d)$ dependent variables, and these variables are independent fair random bits, can be solved in ${\text {poly}}(d)+O(\log \log \log n)$ rounds in deterministic low-space MPC, using $n^{1+o(1)}$ global space and $n^{{\text {poly}}(d)}$ local computation.

##### Proof

We derandomize the LOCAL algorithm of Fischer and Ghaffari [19]. The algorithm consists of an ${\tilde{O}}(d)+\log ^* n$ initial deterministic coloring procedure from [21], an $O(d^2)$-round randomized *pre-shattering* part, and then a ${\text {polyloglog}}(n)$-round deterministic *post-shattering* part (which follows from plugging the network decomposition result of [45] into the derandomization framework of [24] applied to the LLL algorithm of [13]; see [45] for details). We first collect the $O(d^2+{\text {polyloglog}}(n))$-radius ball around each event onto a single machine; these balls are of size $d^{O(d^2+{\text {polyloglog}}(n))} = n^{o(1)}$, and each event’s dependence on its variables can be specified in $2^{{\text {poly}}(d)}=n^{o(1)}$ bits, so the balls fit on single machines. This collection process can be performed in $O(\log d +\log \log \log n)$ rounds by graph exponentiation.

We can now directly and immediately simulate the initial coloring, and we will also be able to directly simulate the deterministic post-shattering part, so it remains to derandomize the pre-shattering part. We do so using hash functions. First, note that the proof of the Shattering Lemma [19, Lemma 6], giving the crucial shattering property of the randomized part, requires independence only between the dependent variables of groups of $O({\text {poly}}(d)\log n)$ events at a time (the *O*(1)-radius neighborhoods of groups of $O(\log n)$ events). So, it requires variables to be sampled from their distributions with only $({\text {poly}}(d) \cdot \log n)$-wise independence.

By Theorem 14, for any constant $c\in {\mathbb {N}}$, there exists a strongly $(n^{-c},{\text {poly}}(d)\log n)$-wise independent family ${\mathcal {H}}$ of functions $[{\text {poly}}(n)]\rightarrow \{0,1\}$ of size $2^{{\text {poly}}(d)\log n}$, i.e., requiring ${\text {poly}}(d)\log n$ bits to specify each function. We use such a function to generate the values taken by all variables. By taking *c* to be sufficiently high, we can ensure that the statistical error of the hash functions in negligible. Since the algorithm of [19] succeeds with high probability in *n* when variables are sampled independently at random, we have that using a uniformly random function from ${\mathcal {H}}$ to provide variable values also causes the algorithm to succeed w.h.p.

We can therefore perform a distributed implementation of the method of conditional expectations to deterministically fix a function which causes the algorithm to succeed. Czumaj et al. [14, 16] show how to implement this method, in low-space MPC, in such a way that $\Theta (\log n)$ bits specifying the function can be fixed in a single round, provided success at any node, under any function from ${\mathcal {H}}$, can be checked locally on a machine. Here, we can check success locally by simply running the algorithm of [19] to completion using the given function, since we have already collected sufficiently large local neighborhoods onto single machines.

We therefore require ${\text {poly}}(d)$ MPC rounds to perform the method of conditional expectations and derandomize the LLL algorithm. So, the total round complexity is ${\text {poly}}(d)+O(\log \log \log n)$. The global space usage is dominated by the storage of an $O(d^2+{\text {polyloglog}}(n))$-radius ball around each node, i.e., $n d^{O(d^2+{\text {polyloglog}}(n))}= n^{1+o(1)}$ global space. The local computation is dominated by the method of conditional expectations, which requires evaluating all functions from ${\mathcal {H}}$, taking $n^{{\text {poly}}(d)}$ computation. $\square$

#### Sinkless orientation

In this section, we combine the lifting of the LOCAL lower bounds to deterministic low-space component-stable MPC algorithms (Theorem 5) with derandomization of the constructive Lovász Local Lemma (Lemma 17) to show that conditioned on the connectivity conjecture, deterministic low-space component-unstable MPC algorithms for sinkless orientation are provably faster than their component-stable counterparts.

We define sinkless orientation to be the problem of orienting the edges of a graph, such that each node of minimum degree at least 3 has at least one outgoing edge (this minimum degree criterion is necessary, since otherwise the problem is not possible, e.g., on a path).

A lower bound for sinkless orientation in the LOCAL model was first proven by [9], and extended to a stronger bound for deterministic algorithms by [12]. When combined with Theorem 5, we obtain the following theorem.

##### Theorem 18

Assuming the connectivity conjecture, there is no deterministic component-stable low-space MPC algorithm that computes a sinkless orientation in $o(\log \log _\Delta n)$ rounds, even in forests.

##### Proof

By [9, 12], there is an $\Omega (\log _\Delta N)$ lower bound for deterministic sinkless orientiation even in $\Delta$-regular forests, and this holds even if nodes have unique $O(\log N)$-bit IDs and know the exact values of *n* and $\Delta$. Since sinkless orientation is an edge-labeling problem, we must work on the line graph of the input graph in order to meet our vertex-labeling framework. We therefore let ${\mathcal {G}}$ be the (normal) class of all line graphs of forests, and, setting $T(N,\Delta ):= \log _{\Delta }^{1/3}N$ (which is a constrained function) we have an $T(N,\Delta )$-round LOCAL lower bound for the vertex-labeling version on line graphs. By Theorem 5, we obtain an $\Omega (\log (T(N,\Delta ))) = \Omega (\log \log _{\Delta } n)$-round lower bound for deterministic low-space MPC algorithms, conditioned on the connectivity conjecture. Converting back from the line graph formulation to the original input graphs, this lower bound is on the family of forests. $\square$

Theorem 18 is complemented by the following result providing a deterministic low-space component-unstable MPC algorithm for sinkless orientation that surpasses the component-stable lower bound for low-space MPC.

##### Theorem 19

There is a deterministic low-space MPC algorithm that computes a sinkless orientation, in any graph of $\Delta =\log ^{o(1)}\log n$ maximum degree, in ${\text {poly}}(\Delta )+O(\log \log \log n) = o(\log \log _\Delta n)$ rounds, using $n^{1+o(1)}$ global space. The algorithm is component-unstable, and uses $n^{{\text {poly}}(\Delta )}$ local computations.

##### Proof

We concentrate on the algorithm for the case of *d*-regular graphs with $d>500$; Ghaffari and Su [27] show how extend to irregular graphs with minimum degree at least 3 using $O(\log ^*n)$ rounds of deterministic pre-processing and *O*(1) rounds of deterministic post-processing in LOCAL, which we can simulate directly in low-space MPC.

In this case, sinkless orientation can be solved by a single application of the distributed LLL (see e.g. [9]), with $d=\Delta$ and $p=2^{-\Delta }$ (setting $d>500$ is more than sufficient to make this instance satisfy the LLL criterion of Fischer and Ghaffari’s algorithm), and the underlying variables are simple uniformly random choices of orientation for each edge (so can be represented with a single fair random bit). Applying Lemma 17, we obtain a ${\text {poly}}(\Delta ) + O(\log \log \log n)$-round deterministic low-space MPC algorithm using $n^{{\text {poly}}(\Delta )}$ local computation. $\square$

We also remark that, for $\Delta \le n^{o(1/\log \log n)}$, an $O(\log \log \log n)$-round component-stable *randomized* algorithm exists for the problem, by simply collecting $\Theta (\log \log n)$-radius balls onto machines via graph exponentiation, and then simulating the randomized LOCAL algorithm of [27]. Sinkless orientiation is therefore an example of a problem with a (conditional) separation between randomized and deterministic algorithms, proving Theorem 8.

#### Edge-coloring

Similarly to the sinkless orientation problem, the framework combining the lifting of the LOCAL lower bounds to deterministic low-space component-stable MPC algorithms (Theorem 5) with derandomization of the constructive Lovász Local Lemma (Lemma 17) can be used to show that assuming the connectivity conjecture, for the classical edge-coloring there are deterministic low-space component-unstable MPC algorithms that are provably faster than their component-stable counterparts. We begin with the following application of Theorem 5.

##### Theorem 20

Assuming the connectivity conjecture, there is no deterministic component-stable low-space MPC algorithm that computes a $(2\Delta -2)$-edge coloring, even in forests, in $o(\log \log _\Delta n)$ rounds.

##### Proof

Chang et al. [11] give a deterministic LOCAL lower bound of $\Omega (\log _\Delta N)$ for $(2\Delta - 2)$-edge coloring, even in forests. To fit $(2\Delta - 2)$-edge coloring into our framework (in which problem outputs are labels on nodes) we must define it as vertex-coloring on the line graph. In LOCAL, operations on the line graph can be simulated in the original graph and vice versa with only one round additive overhead. So, we have an $\Omega (\log _\Delta N)$-round deterministic lower bound for ‘$(2\Delta - 2)$-coloring the line graphs of forests with maximum degree $\Delta$’.

The family of line graphs of forests is both hereditary (deleting a node in the line graph corresponds to deleting an edge in the original graph, and forests are closed under edge deletion) and closed under disjoint union (which corresponds to disjoint union in the original graph), so it is normal under Definition 2. We set $T(N,\Delta ):=\log ^{1/3}_\Delta N$, a constrained function. Then, by Theorem 5, we obtain a conditional $\Omega (\log \log _\Delta n)$ deterministic lower bound for $(2\Delta - 2)$-edge coloring forests with *n* nodes and maximum degree $\Delta$. $\square$

##### Theorem 21

There is a deterministic low-space MPC algorithm that computes a $(\Delta +\sqrt{\Delta }\log ^3 \Delta )$-edge coloring, in any graph of maximum degree $\Delta =\log ^{o(1)}\log n$, in $O({\text {poly}}(\Delta )+\log \Delta \log \log \log n)= o(\log \log _{\Delta } n)$ rounds, using $n^{1+o(1)}$ global space. The algorithm is component-unstable and uses $n^{{\text {poly}}(\Delta )}$ local computation.

##### Proof

We apply the algorithm of [11]. Setting $\varepsilon =\frac{\log ^3 \Delta }{\sqrt{\Delta }}$ in Theorem 4 of [11] gives a running time dominated by $O(\log \Delta )$ applications of LLL, with $d = {\text {poly}}(\Delta )$ and $p=\Delta ^{-\omega (1)}$. Furthermore, the variables in the LLL instances are uniformly random choices of colors from each edge’s palette (of some current palette size *P*). These can be generated from ${\text {poly}}(d)$ fair random bits in such a way that the probability of choosing any particular color differs from 1/*P* by at most $2^{-{\text {poly}}(d)}$; the probability of each bad event therefore also incurs error of $2^{-{\text {poly}}(d)}$, and so remains $\Delta ^{-\omega (1)}$.

Applying Lemma 17, we can perform all applications of LLL in $O({\text {poly}}(\Delta )+\log \Delta \log \log \log n)$ rounds in deterministic low-space MPC with $n^{1+o(1)}$ global space. The algorithm of [11] then applies a final $5\Delta '$-edge coloring algorithm to finish off the remaining graph (where $\Delta '$ is the maximum degree of this graph); we can do this in $O(\log ^* n)$ rounds by simulating Linial’s (deterministic) vertex-coloring algorithm [37] on the line graph. The total round complexity is $O({\text {poly}}(\Delta )+\log \Delta \log \log \log n)$, the global space is $n^{1+o(1)}$, and the local computation is $n^{{\text {poly}}(\Delta )}$. $\square$

#### Vertex-coloring triangle-free graphs

A combination of Theorem 5 and Lemma 17 can be used to show a similar complexity gap between deterministic component-stable and component-unstable MPC algorithms for vertex coloring.

##### Theorem 22

Assuming the connectivity conjecture, there is no deterministic component-stable low-space MPC algorithm that computes a $\Delta$-vertex coloring, even in forests, in $o(\log \log _\Delta n)$ rounds.

##### Proof

Chang et al. [12] give an $\Omega (\log _{\Delta } N)$-round deterministic lower bound for $\Delta$-coloring trees (and therefore also the normal class of *forests*) of maximum degree $\Delta$ (for $\Delta \ge 3$). Setting $T(N,\Delta ):=\log ^{1/3}_\Delta N$ (a constrained function), by Theorem 5, we obtain a conditional $\Omega (\log \log _\Delta n)$ deterministic lower bound for $\Delta$-coloring forests with *n* nodes and maximum degree $\Delta$. $\square$

##### Theorem 23

There is a deterministic low-space MPC algorithm that computes a $O(\frac{\Delta }{\log \Delta })$-vertex coloring, in any triangle-free graph of $\Delta =\log ^{o(1)}\log n$ maximum degree, in ${\text {poly}}(\Delta ) + O(\log \Delta \log \log \log n) = o(\log \log _{\Delta } n)$ rounds, using $n^{1+o(1)}$ global space. The algorithm is component-unstable and uses $n^{{\text {poly}}(\Delta )}$ local computations.

##### Proof

We plug in our LLL algorithm into the algorithm of [43]. When $\Delta = o(\log n)$, this algorithm consists of $O(k+\log ^* n)$ applications of LLL in order to $O(\frac{\Delta }{k})$-color the graph, where $k \le (\frac{1}{4} - o(1))\ln \Delta$. We choose some such $k=\Theta (\log \Delta )$.

Similarly to the proof of Theorem 21, the LLL instances required have $d = {\text {poly}}(\Delta )$ and $p=\Delta ^{-\omega (1)}$, and the variables are uniformly random color choices, which we can generate from ${\text {poly}}(d)$ fair coins while only increasing the probability of any bad event by $2^{-{\text {poly}}(d)}$. So, we can apple Lemma 17, and obtain a deterministic low-space MPC algorithm for $O(\frac{\Delta }{\log \Delta })$-coloring triangle-free graphs in $O({\text {poly}}(\Delta ) +\log \Delta \log \log \log n)$ rounds, using $n^{1+o(1)}$ global space, with $n^{{\text {poly}}(\Delta )}$ local computation. $\square$

For all the problems above, sinkless orientation, edge-coloring, and vertex-coloring, we have obtained component-unstable algorithms which surpass the conditional lower bounds for component-stable algorithms when $\Delta = \log ^{o(1)}\log n$. Furthermore, though in general these algorithms use heavy local computation, for bounded degree ($\Delta = O(1)$) graphs their local computation is ${\text {poly}}(n)$. Since we still surpass the lower bounds for sufficiently large constant $\Delta$, this demonstrates that *component-instability helps for deterministic algorithms even using polynomial computation*.

### Extendable algorithms

We next describe an explicit derandomization recipe for a particular class of LOCAL algorithms. This recipe allows one to derandomize *r*-round local algorithms within $O(\log r)$ low-space MPC rounds, provided that the *r*-radius ball of each node in the graph *G* fits the local space of the machines. As a consequence, we show component-unstable algorithms for maximal independent set and maximal matching that surpass the lower bounds for component-stable algorithms.

We call the class of LOCAL algorithms we consider *extendable*. Roughly speaking, these algorithms can extend any partial legal solution (e.g., a collection of legally colored nodes) into a complete solution (similar to the notion of *greedy* algorithms). In addition, the local computation performed at each node in these algorithms must be polynomial. Even though the LOCAL model does not account for the local computation time, most of the LOCAL algorithms are in fact efficient in this regard. We next define this notion more formally.

#### Definition 17

(*Extendable algorithms*) Let ${\mathcal {A}}$ be a randomized LOCAL algorithm for an LCL problem ${\mathcal {P}}$ with round complexity at most $T(n,\Delta )$ (where *T* is non-decreasing in *n* and $\Delta$) on every *n*-node graph, when provided with the exact value of *n* and maximum degree $\Delta$. Then, ${\mathcal {A}}$ is **extendable** if:

(i) Any partially decided subgraph can be extended into a global solution for ${\mathcal {P}}$. Formally, ${\mathcal {A}}$ returns an output labeling on *G* giving each node a label in $L\cup \{\bot \}$, where *L* is the output alphabet permitted by the problem ${\mathcal {P}}$. Then, re-labeling the nodes labeled $\bot$ with any valid output labeling on their induced graph must give a valid output labeling on *G*. This must hold with certainty (even though ${\mathcal {A}}$ is a randomized algorithm).
(ii) In expectation, ${\mathcal {A}}$ labels less than $\frac{1}{2}$ nodes $\bot$.
(iii) In every round *i* of ${\mathcal {A}}$, each node *u* performs at most $\Delta ^{O(T(n,\Delta ))}$ internal computation to determine its output for that round (e.g., the messages to be sent in the next round, internal states, and its final output in the last round).

We next show that using the PRG construction of Lemma 16 any extendable LOCAL algorithm running in $t=T(n,\Delta )$ rounds can be simulated deterministically within $O(\log t)$ MPC rounds. This has various applications for derandomizing LOCAL algorithms on low-degree graphs, which consequently yields a separation between stable and unstable deterministic MPC algorithms. For simplicity, we consider LOCAL algorithms for LCL problems, however, this can be extended for the approximation variants of LCL problems (e.g., approximate max-IS and maximal matching).

#### Theorem 24

For every constant $\phi \in (0,1)$, there is some constant $\gamma$ such that any $T(n,\Delta )$-round extendable LOCAL algorithm ${\mathcal {A}}$ satisfying that $\Delta ^{\gamma \cdot T(n,\Delta )} \le n^{\phi }$, in which each node uses at most $\Delta ^{O(T(n,\Delta ))}$ bits of randomness, can be derandomized,^13^ within $O(\log (T(n,\Delta ))+\log ^*n)$ MPC rounds with local space $O(n^\phi )$ and global space $O(n^{1+\phi })$.

#### Proof

Consider a fixed *n*-node graph $G=(V,E)$, with maximum degree $\Delta$, and let $t:=T(n,\Delta )$. First, the MPC algorithm allocates a separate machine $M_u$ to each node *u* that stores its 2*t*-radius ball in *G*. This can be done in $O(\log t)$ rounds, by the standard graph exponentiation technique. Next, the algorithm computes a $\Delta ^{4t}$-coloring in the graph $G^{2t}$. The purpose of this step is to reduce the name space of the nodes from $O(\log N)$ bits into $O(t \log \Delta )$ bits, such that in each 2*t*-radius ball, the new IDs of the nodes (i.e., their colors) are unique. This coloring can be implemented within $O(\log ^* N)$ deterministic rounds [33].

We will simulate *O*(1) iterations of algorithm ${\mathcal {A}}$, each time running on the subset of nodes labeled with $\bot$ in the last iteration. That is, we start on our input graph *G*, and provide ${\mathcal {A}}$ with the values *n* and $\Delta$, and in subsequent iterations provide the current values $n^*\le n$ and $\Delta ^* \le \Delta$. By extendability of ${\mathcal {A}}$, we know that the resulting labeling can be extended to a valid full solution by relabeling $\bot$ with any valid labeling on their induced graph. It remains only to show that after *O*(1) iterations, we can ensure that *no* nodes remain labeled $\bot$.

In each iteration of ${\mathcal {A}}$ we let machine $M_u$ determine the output for node *u*, and we use the PRG construction of Lemma 16 in order to deterministically fix a good random seed for the algorithm. We need to provide each node with $\Delta ^{O(t)}$ random bits, and we do so based on the new IDs (i.e., nodes with the same new IDs receive the same random bits, but since these nodes are of distance at least 2*t* apart, this does not cause any dependency issues). So, our PRG will need to generate $\Delta ^{O(t)}$ total pseudorandom bits.

By Lemma 16, a $(\Delta ^{O(t)}, n^{-\varepsilon })$ PRG can be constructed using $e^{O(t\log \Delta +\varepsilon \log n)}$ space. Setting $\varepsilon$ to be a sufficiently small constant, this is $O(n^\phi )$. Note that we always use this PRG (i.e., with parameters in terms of original number of nodes *n*), and do not update to the current value $n^*$.

When we run ${\mathcal {A}}$, with a uniformly random seed from the PRG, on a graph of size $n_*\le n$ (since we run only on the induced graphs of subsets of nodes labeled $\bot$), the output at each node is $(\Delta ^{O(t)}, n^{-\varepsilon })$ indistinguishable from its output under full randomness. So, the expected number of nodes which output $\bot$ is at most $\frac{1}{2}+n_*\cdot n^{-\varepsilon }$. After $O(1/\varepsilon )$ iterations we therefore reduce the expected number of nodes labeled $\bot$ below 1.

Now it remains only to *deterministically* choose a seed from the PRG that achieves this expected value of undecided nodes (and, since the number of undecided nodes is an integer, it must then be 0). To do so, we use a distributed implementation of the classical method of conditional expectations, a means of having all machines agree on some seed which achieves at most the expected value of some cost measure (in this case, the number of nodes labeled $\bot$). Czumaj et al. [14, 16] show how to implement this method, in low-space MPC, in such a way that $\Theta (\log n)$ bits specifying the seed can be fixed in a single round, provided that the global cost measure is the sum of functions computable locally on single machines. Here, each machine $M_u$ can locally compute the indicator variable for the event $\{ u \hbox {is labeled} \bot \}$ under ${\mathcal {A}}$ using any particular seed, and the global cost function is the sum (over all nodes $u\in V$) of these variables.

Since the seeds for the PRG are $O(t\log \Delta +\varepsilon \log n) = O(\log n)$ bits long, we can perform the method of conditional expectations in *O*(1) rounds in each iteration, fixing a globally agreed seed from our PRG which ensures that the number of nodes labeled $\bot$ is at most its expectation. Then, after $O(\frac{1}{\varepsilon }) = O(1)$ iterations, we have no remaining nodes labeled $\bot$, and so have a complete valid solution for the problem. The total running time of the deterministic MPC algorithm is therefore $O(\log t)$ (for initially collecting balls) plus $O(\log ^* n)$ (for coloring). $\square$

#### Application to maximal independent set and maximal matching

To demonstrate the applicability of this derandomization recipe, we show how it can be used to improve the deterministic running times of two cornerstone problems in low-space MPC: maximal independent set and maximal matching. The best prior round complexities for both problems is $O(\log \Delta +\log \log n)$ [14]; here we improve the dependency on *n* to triple-logarithmic, and surpass the component-stable lower bound for $\Delta =2^{\log ^{o(1)} n}$.

##### Theorem 25

For any constant $\phi > 0$, a maximal independent set and maximal matching can be found deterministically in low-space MPC in $O(\log \log \Delta + \log \log \log n)$ rounds when $\Delta =2^{\log ^{o(1)} n}$, with local space $O(n^\phi )$ and global space $O(n^{1+\phi })$.

##### Proof

We focus on maximal independent set, since the results for maximal matching will then follow by reduction.

We will use the LOCAL algorithm of Ghaffari [22], which, when combined with the polylogarithmic network decomposition of [45], runs in $t = O(\log \Delta + {\text {polyloglog}}(n))$ rounds, on graphs with *n* nodes, when provided with the values *n* and $\Delta$, succeeding (globally) with probability at least $1-\frac{1}{n^2}$. We use the following final labeling: nodes which have been placed in the output independent set are labeled $\mathbb{I}\mathbb{N}$, adjacent nodes are labeled $\mathbb {OUT}$, and all other nodes are labeled $\bot$.

Ghaffari’s algorithm has the following important property: even when the algorithm probabilistically fails, it never places two adjacent nodes in the output independent set; instead it merely fails to decide the status of some nodes. Therefore, any output labeling produced by Ghaffari’s algorithm in this way is extendable to a full solution by any valid output (i.e., a valid MIS) on the induced graph of undecided nodes (those labeled $\bot$).

Another property we need is a bound on the number of random bits used by each node. Ghaffari’s algorithm uses $O(t \log \Delta )$ random bits per node ($O(\log \Delta )$ per round *i*), since a node *v*’s only random choice each round is to ‘mark’ itself with some probability $p_t(v)$, which is equal to $2^{-k_i}$ for some $k_i\in [\lceil \log \Delta \rceil ]$. To perform this choice, *v* can take $k_i$ random bits and mark itself if they are all 0.

Furthermore, since with probability at least $1-\frac{1}{n^2}$ the algorithm succeeds globally (i.e., labels no nodes $\bot$), the *expected* number of nodes labeled $\bot$ is at most $\frac{n}{n^2}<\frac{1}{2}$.

Therefore the algorithm is extendable. We have $\Delta ^{O(t)} = 2^{\log ^{o(1)} N} \le N^{\phi }$, and so we can apply Theorem 24 to solve MIS in $O(\log t) = O(\log \log \Delta +\log \log \log n)$ rounds deterministically in MPC with local space $O(n^\phi )$ and global space $O(n^{1+\phi })$.

To perform Maximal Matching, we use standard reduction of finding an MIS on the line graph of the input graph. It is well-known that this corresponds to a maximal matching in the original graph, and in LOCAL only increases the round complexity by 1, which means that we still adhere to our MPC space bounds. $\square$

##### Theorem 26

Assuming the connectivity conjecture, there is no deterministic low-space component-stable MPC algorithm that computes a maximal matching or maximal independent set in $o(\log \Delta +\log \log n)$ rounds.

##### Proof

Balliu et al. [5] show an $\Omega (\min \{\Delta ,\frac{\log N}{\log \log N}\})$-round^14^ deterministic LOCAL lower bound for both problems, even when exact values of *n* and $\Delta$ are known. Using constrained function $T(N,\Delta ) = \sqrt{\min \{\Delta ,\log N\}}$, we apply Theorem 5 to obtain an $\Omega (\min \{\log \Delta ,\log \log N\})$-round conditional lower bound for component-stable low-space deterministic MPC algorithms, (directly for maximal independent set, and using the standard conversion to the line graph for maximal matching). $\square$

Hence, Theorem 25 surpasses this component-stable conditional lower bound when $\Delta =2^{\log ^{o(1)} n}$.

In earlier versions of this work, as a corollary of Theorem 25, we gave a round complexity of $O(\log \Delta + \log \log \log n)$ rounds for maximal independent set and maximal matching, in MPC with local space $n^\phi$ and global space $n^{1+\phi }$. While this is indeed achievable, we note that there is in fact a straightforward way in which to achieve an even faster round complexity:

##### Lemma 27

For any constant $\phi > 0$, a maximal independent set and maximal matching can be found deterministically in low-space MPC in $O(\log \Delta + \log (\log ^* n))$ rounds, with local space $n^\phi$ and global space $n^{1+\phi }$.

##### Proof

When $\Delta =\Omega (\root 3 \of {\log n})$, we use the low-space MPC algorithm of [14], with running time $O(\log \Delta +\log \log n) = O(\log \Delta )$. When $\Delta =o(\root 3 \of {\log n})$, we collect balls of radius $O(\Delta ^2+\log ^* n)$ around each node onto dedicated machines. This takes $O(\log \Delta +\log (\log ^* n))$ rounds via graph exponentiation, and does not exceed the space bounds since the total size of such balls is $\Delta ^{O(\Delta ^2+\log ^* n)} = (\log n)^{o(\log ^{2/3} n+\log ^* n)} = n^{o(1)}$. Machines can then immediately simulate the deterministic distributed maximal independent set and maximal matching algorithms of [42] (which require knowledge of the $O(\Delta ^2+\log ^* n)$-ball and $O(\Delta +\log ^* n)$-ball respectively) to obtain the output for their dedicated node. $\square$

## Separation between stable and unstable randomized MPC

In this section, we demonstrate the existence of a natural problem for which there is a (conditional) gap between *randomized* component-stable and component-unstable algorithms. This will give us a proof of Theorem 1.

We consider the task of computing large independent sets in *n*-node graphs with maximum degree $\Delta$. Recently, [32] has shown that for any *n*, there exists *n*-node graphs with maximum degree $\Delta = \Omega (n/\log n)$, for which any randomized LOCAL algorithm for computing an independent set of size $\Omega (n/\Delta )$ with success probability $1-\frac{1}{n}$ (in fact, even reaching a weaker success guarantee of $1-\frac{1}{\log n}$), requires $\Omega (\log ^*n)$ rounds. (Here, and throughout this section, we assume that $\Delta \ge 1$ so that the problem is well-defined.) Since the cardinality of the maximum independent set in their lower bound graph can be bounded by $O(n/\Delta )$, their result can also be stated as a lower bound for computing a constant approximation for the maximum independent set. We start by providing a mild adaptation to the lower bound proof of [32] so that it would fit the framework from Sect. 2 and from [26]. In particular, to be able to lift this LOCAL lower bound into the MPC setting from Theorem 5 in Sect. 2 and from [26], it is required for the lower bound to hold even if nodes are equipped with shared randomness, and with an estimate *N* on the number of nodes *n* which is at least more than a $\log N$-factor loose, i.e., nodes cannot distinguish between $n=\Theta (N/\log N)$ and $n=\Theta (N)$. We begin with the following fact implicit in Corollary V.4 of [26].

### Fact 1

[26] Any randomized LOCAL algorithm to compute a maximum independent set in *n*-node cycle graph requires $\Omega (\log ^*n)$ rounds; this holds even if the nodes know *n* (and, naturally, know $\Delta = 2$) and even if they have access to an unlimited amount of shared randomness.

We can then plug this lower bound, strengthened to hold against shared randomness, into the result of [32], yielding Theorem 28. The proof is identical to Theorem 4 of [32]; it is unaffected by the change to shared randomness.

### Theorem 28

[32] Any randomized LOCAL algorithm to compute an independent set of size $\Omega (n/\Delta )$ in any *n*-node graph (i.e., over the full range of $\Delta \in [1,n)$) requires $\Omega (\log ^* n)$ rounds. This holds even if the nodes have the exact maximum degree $\Delta$, access to an unlimited amount of shared randomness, and an input size estimate *N* of *n* which is more than a $\log N$-factor loose, i.e., they cannot distinguish between $n=\Theta (N/\log N)$ and $n=\Theta (N)$.

We can now combine Theorem 28 and Lemma 3 with the lifting argument in Theorem 5, using the constrained function $T(N,\Delta ):= \log ^* N$, to obtain the following lower bound for component-stable MPC algorithms.

### Lemma 29

(Super-constant lower bound for component-stable IS) Assuming that the connectivity conjecture holds, there is no $o(\log \log ^* n)$-round low-space component-stable MPC algorithm that computes an independent set of size $\Omega (n/\Delta )$, in any graph with *n* nodes and $\Delta \in [1, n)$, with success probability at least $1-\frac{1}{n}$.

Finally, we show that there is a simple component-unstable MPC algorithm for computing large independent sets in a constant number of rounds.

### *O*(1)-round randomized algorithm

We first give a *O*(1)-round randomized algorithm that computes an independent set of $\Theta (n/\Delta )$ nodes, in expectation. We can then apply derandomization techniques to reach an algorithm that does so with certainty. First, each node computes its degree, which can be done in *O*(1) rounds. The algorithm then consists of a single step of Luby’s algorithm (see [38]), where each node *v* picks a number $\chi _v \in [0,1]$ uniformly at random. A node *v* joins the independent set if $\chi _v < \chi _u$ for every neighbor *u* of *v*. This can be verified in *O*(1) rounds in the low-space MPC setting. We next observe that the probability that a node *v* has the minimum $\chi _v$ value in its neighborhood is at least $1/(d_v+1) \ge 1/(\Delta +1)$. Thus, in expectation, the number of nodes that joins the independent set is at least $n/2\Delta$.

### *O*(1)-round deterministic algorithm

We slightly change the algorithm described above so that it would work with pairwise independence, and hence to become deterministic.

#### Claim 1

Consider a simulation of a single step of Luby’s algorithm with pairwise independence. Then, the expected number of independent set nodes is $n/4\Delta$.

#### Proof

For a node *v* we consider the event that $\chi _v < 1/(2\Delta )$ and that $\chi _u \ge 1/(2\Delta )$ for every neighbor *u* of *v*. Note that if this event occurs then *v* joins the independent set. To bound the probability of this event for node *v*, we first bound the probability that $\chi _u \ge 1/(2\Delta )$ for every neighbor *u* *conditioned* on the event that $\chi _v < 1/(2\Delta )$. Due to pairwise independence, for every neighbor *u*, it holds that $\chi _u \le 1/(2\Delta )$ with probability $1/2\Delta$ even when conditioning on $\chi _v < 1/(2\Delta )$. Thus, conditioned on $\chi _v < 1/(2\Delta )$, by the union bound, the probability that *v* has some neighbor $u'$ with $\chi _{u'} \le 1/2\Delta$ is at most 1/2. Overall, the probability for the event to hold is $1/4\Delta$. $\square$

This step can be derandomized by applying *O*(1) steps of graph sparsification, in a similar manner as done for the derandomization of Luby’s step in the maximal independent set algorithm of [14], yielding the following:

#### Theorem 30

There is a deterministic low-space MPC algorithm that in *O*(1) rounds computes an independent set of size $\Omega (n/\Delta )$, in any graph on *n* nodes with $\Delta = [1,n)$.

#### Proof

Let $\delta >0$ be a constant sufficiently smaller than $\phi$. If $\Delta > n^{2\delta }$, we perform an initial derandomized sparsification procedure following the lines of [14]. Specifically, we proceed in $j:=\lceil \frac{\log \Delta }{\delta \log n}\rceil -2$ ($=O(1)$) iterations. Each iteration is based on the randomized process of having each node remain in the graph with probability $n^{-\delta }$, and drop out otherwise. As in [14], we can show that, even if the random choices are only *c*-wise independent for some sufficiently large constant *c*, with high probability the maximum degree in the graph reduces by a factor of $\Omega (n^{\delta })$. Furthermore, the *expected* number of nodes in the graph reduces by a factor of $n^{-\delta }$. So, we can use a random function from a *c*-wise independent family of hash functions for the randomness (taking $O(\log n)$ bits to specify), and then fix such a function for which the above two properties hold (i.e. maximum degree reduces by $\Omega (n^{\delta })$, and number of nodes reduces by $O(n^{\delta })$.

Then, we have that after iteration *j*, the number of nodes in the graph is $\Omega (n^{1-j\delta })$, and the maximum degree is $O(\Delta n^{-j\delta }) = O(\Delta n^{(2-\frac{\log \Delta }{\delta \log n})\delta }) = O(2n^\delta )$.

Since degrees are now low enough to fit 2-hop neighborhoods onto single machines (as $\delta$ is sufficiently lower than $\phi$), we can derandomize a step of Luby’s algorithm on this sparsified graph exactly as in [14]: by Claim 1 one step of Luby’s algorithm yields an independent set of *expected* size $\Omega (n'/\Delta ')$ on an $n'$-node graph of maximum degree $\Delta '$, even under only pairwise independent random choices. By applying the method of conditional expectations combined with a pairwise-independent family of hash functions, we can deterministically find an independent set of at least the expected size, in this case $\Omega (\frac{n^{1-j\delta }}{\Delta n^{-j\delta }}) = \Omega (\frac{n}{\Delta })$.

If, instead, we initially have $\Delta \le n^{2\delta }$, then we omit the sparsification procedure and derandomize a step of Luby’s algorithm directly on the input graph, which again yields an independent set of size $\Omega (\frac{n}{\Delta })$. $\square$

## General non-uniform and non-explicit derandomization of MPC algorithms

The conditional lifting arguments for *component-stable* MPC algorithms in Theorem 5 imply that for some (*O*(1)-replicable, and hence, e.g., LCL s) graph problems there is a (conditional) exponential gap between randomized and deterministic algorithms, e.g., for problems that admit such an exponential gap in the LOCAL model [11, 12]. In this section we provide a proof of Theorem 9, showing that in the MPC model with polynomially many machines, no such gap exists from a complexity perspective. That is, we show that any randomized low-space MPC algorithm with round complexity $T(n,\Delta )$ and which uses a polynomial number of machines, can be transformed into a deterministic MPC algorithm that runs in $O(T(n,\Delta ))$ rounds and also uses a polynomial number of machines. This deterministic algorithm is component-unstable, and it is also non-uniform and non-explicit.

We begin with an auxiliary lemma that adapts the approach for LOCAL algorithms from [12] (see also [25]) to MPC algorithms. (Whereas in [12, 25] this claim holds only for LCL problems, in our case we will use it in Lemma 32 for any problem whose solution can be verified efficiently in the MPC setting.)

### Lemma 31

(Implicit in [12]) Let ${\mathcal {A}}$ be (a possibly non-uniform) randomized MPC algorithm that solves a graph problem ${\mathcal {P}}$ on *n*-node graphs with maximum degree $\Delta$ in $T(n,\Delta )$ rounds, with probability at least $1-2^{-n^2}$. Then, there is a *non-uniform*, *non-explicit* deterministic MPC algorithm ${\mathcal {A}}'$ that solves ${\mathcal {P}}$ on *n*-node graphs with maximum degree $\Delta$ in $O(T(n,\Delta ))$ rounds.

The local and global space of algorithm ${\mathcal {A}}'$ is asymptotically the same as that of ${\mathcal {A}}$.

### Proof

As we defined it in Sect. 2.4.4, the randomized MPC algorithm ${\mathcal {A}}$ has access to shared randomness ${\mathcal {S}}$ of polynomial length. Once the string ${\mathcal {S}}$ is fixed, ${\mathcal {A}}$ is deterministic. Let ${\mathcal {G}}_{n,\Delta }$ be the family of graphs with at most *n* nodes and maximum degree $\Delta$. Notice than $\vert {\mathcal {G}}_{n,\Delta } \vert \le 2^{n^2}$. Therefore, if we run ${\mathcal {A}}$ on each seed and each possible seed fails on at least one graph in ${\mathcal {G}}_{n,\Delta }$, the success guarantee of the algorithm ${\mathcal {A}}$ cannot be better than $1-1/2^{n^2}$. So, since ${\mathcal {A}}$ succeeds with probability at least $1-1/2^{n^2}$, there must be at least one seed ${\mathcal {S}}^*$ that when provided to the MPC algorithm, the algorithm is *correct for every graph* in ${\mathcal {G}}_{n,\Delta }$.

This gives us a deterministic algorithm ${\mathcal {A}}'$, which uses the seed ${\mathcal {S}}^*$ to solve ${\mathcal {P}}$ on every graph in ${\mathcal {G}}_{n,\Delta }$. Note that the resulting deterministic algorithm is non-explicit in the sense that the desired seed should be hard-coded into the machines. Furthermore, it is non-uniform in the sense that a different seed is hard-coded for each *n*, and so we must use a different algorithm for each *n* (it is not possible to hard-code the seeds for all *n* into the *same* algorithm, since this would require unbounded space and therefore would not fit on machines, or even over our *poly*(*n*) global space bound). $\square$

### Lemma 32

Let ${\mathcal {A}}$ be (a possibly non-uniform) randomized MPC algorithm that solves a graph problem ${\mathcal {P}}$ on *n*-node graphs with maximum degree $\Delta$ in $T(n,\Delta )$ rounds, with probability at most $1-\frac{1}{n}$ using $n^\alpha$ local space and $n^{\beta }$ global space. In addition, assume that the correctness of the algorithm can be checked in $T(n,\Delta )$ MPC rounds, using $n^\alpha$ local space and $n^{\beta }$ global space. Then, there exists a component-unstable, non-uniform, non-explicit deterministic MPC algorithm ${\mathcal {A}}'$ that solves ${\mathcal {P}}$ on *n*-node graphs with maximum degree $\Delta$ in $O(T(n,\Delta ))$ rounds. The local space of ${\mathcal {A}}'$ is $n^{\alpha }$ and the global space is $\tilde{O}(n^{2+\beta })$.

### Proof

We first boost the success guarantee of ${\mathcal {A}}$ by running multiple parallel simulations: we will run $\ell = O(n^2)$ simulations of algorithm ${\mathcal {A}}$ in parallel, using a distinct set of machines for each simulation. We can determine the correctness of all simulations in $T(n,\Delta )$ MPC rounds, and the final output is determined by choosing an arbitrary correct simulation, if one exists. The probability that the algorithm fails in all these simulations is at most $(\frac{1}{n})^{\ell } = 2^{-\omega (n^2)}$. Thus, if we incorporate this algorithm in Lemma 31, there exists a non-uniform, non-explicit deterministic MPC algorithm ${\mathcal {A}}'$ that solves the problem in $T(n,\Delta )$ rounds using the same local space as ${\mathcal {A}}$ and ${\tilde{O}}(n^{2+\beta })$ global space.

Let us finally mention that the obtained algorithm ${\mathcal {A}}'$ is component-unstable, since it relies on globally agreeing on the outcome of all simulations. $\square$

Now Lemma 32 yields the proof of Theorem 9; informally, $\textsf {DetMPC} = \textsf {RandMPC}$ holds in the regime of polynomially many machines and for non-uniform, non-explicit low-space MPC algorithms.

## Conclusions

In this paper, we investigate the power of component-instability for solving local graph problems in the low-space MPC model. Our main conclusion is that component instability is useful mainly in two related aspects: amplification of the success guarantee and derandomization. In the context of randomized algorithms, it allows one to boost the success guarantee of the algorithm. This appears to be useful especially for approximation problems (e.g., maximizing or minimizing a subset of edges or vertices with a given property). In the context of derandomization, it allows one to efficiently simulate the randomized local algorithm while globally foraging for a short seed. Amplification and derandomization are both obtained by a global computation regardless of the connected components of the graph.

We have shown a variety of problems for which component-instability provides additional power in low-space MPC, and allows algorithms to surpass the component-stable conditional lower bounds given by Ghaffari et al. [26] and revised and extended here. We note, however, that we know of no examples of randomized LCL problems for which this is the case. Indeed, the ways in which we have exploited component-instability would not seem to help for such problems. A major open problem left by this work is therefore to either provide such an example, or to provide conditional hardness results for randomized LCL problems that hold also for unstable algorithms.

## Footnotes

1 By global problems we mean those that require communication across entire connected components to solve, and so have $\Theta (n)$ worst-case LOCAL complexity, as opposed to local problems that have *o*(*n*) LOCAL complexity.

2 The original lifting arguments of [26] only hold for LOCAL lower bounds that hold under exact knowledge of *n*; the lower bound of [32] does not, but holds only under knowing a polynomial estimate of *n*, which is allowed for in our framework.

3 Indeed, this causes an issue with the proof of Lemma III.1 of [26], where success probability amplification is used in an algorithm $A_{\textsf {MPC}}$ that is later (in Lemma IV.2 in [26]) assumed to be component-stable.

4 Specifically in proof of [26, Lemma IV.2] where algorithm $A_{MPC}$ is simulated on large simulation graphs containing smaller components *G* and $G'$, as discussed above, its behavior on *G* and $G'$ as components is only identical to when run on them as sole input if no dependency on *n* is permitted.

5 This sum is calculated by specifying a constant-depth, $n^\varepsilon$-ary tree over the machines, for some sufficiently small constant $\varepsilon$, and then aggregating from the leaves to the root of the tree, summing at every step. For more details on basic MPC operations such as searching and sorting, see e.g. [29].

6 Note that, unlike the changes regarding dependency on *n* and problem class, this change is not necessary to show a general framework for conditional MPC lower bounds—the same results could be proven assuming fully-unique IDs (at least for randomized algorithms) using techniques from [26]. However, we feel that this definition better captures the ‘spirit’ of component-stability.

7 We will define component-stable outputs to not depend on the names of machines—this is not a major restriction, since we are not aware of any MPC algorithms which are not independent of renaming machines. However, it is an important point here, since $B_{MPC}$’s machine names now depend on node names, upon which $B_{MPC}$’s output must not depend.

8 *r*-radius graphs are, by definition, connected, so component-unique IDs are unique IDs.

9 The reason we assume that only a polynomial estimate *N* of *n* is known here is that some LOCAL lower bounds to which we wish to apply our lifting result only hold without exact knowledge of *n* (e.g., that of [32]). Most LOCAL lower bounds, however, do hold under exact knowledge, and in these cases we can simply set $N=n$.

10 When $n_H \ge 2$; for $n_H \le 1$ Lemma 10 is trivial since there can be no LOCAL lower bounds on such graphs.

11 As a counterexample, let *T*(*N*) be a tower of 2 s of height $\log ^*N - 3$. Then, $T(N) = O(\log \log N)$, but $\limsup \limits _{N\rightarrow \infty }\frac{T(N+1)}{T(N)} = \infty$.

12 Lemma 10 shows the *existence* of such graphs, but a uniform MPC algorithm must also be able to *find* them (a non-uniform algorithm can simply have them ‘hard-coded’ in). To do so, we can run a brute-force search on each machine; $O(n^{\phi })$ space is required to identify each of the $2^{O(n^{\phi })}$ pairs of graphs and check the sensitivity property. However, we require $2^{O(n^{\phi })}$ local computation (which is also implicitly the case in [26]). For specific lower bounds there may be efficient constructions, but this must be determined on a case-by-case basis.

13 The deterministic MPC algorithm in the derandomization relies on the PRG construction in Lemma 16, and therefore it may perform *heavy local computations* or alternatively, this result can be stated as a *non-uniform derandomization* where the PRG function is hard-coded in the MPC machines, in which case local computation is polynomial.

14 That is, a deterministic LOCAL algorithm cannot simultaneously have round complexity $o(\Delta )$ and $o(\frac{\log N}{\log \log N})$; algorithms do exist which are faster in one parameter at the expense of the other.
